# Supplementary material for: Pyridylpiperazine efflux pump inhibitor boosts in vivo antibiotic efficacy against K. pneumoniae
Source: EMBO Mol Med. 2023 Dec 20;16(1):93–111. doi: 10.1038/s44321-023-00007-9 (PMC10897476; doi:10.1038/s44321-023-00007-9)

Appendix

Pyridylpiperazine efflux pump inhibitor boosts *in vivo* antibiotic efficacy against *K. pneumoniae*

Anais Vieira Da Cruz[1#], Juan-Carlos Jiménez-Castellanos[2#], Clara Börnsen[3#], Laurye Van Maele[2#], Nina Compagne[1], Elizabeth Pradel[2], Reinke T. Müller[4], Virginie Meurillon[1], Daphnée Soulard[2], Catherine Piveteau[1], Alexandre Biela[1], Julie Dumont[1], Florence Leroux[1,5], Benoit Deprez[1], Nicolas Willand[1], Klaas M. Pos[4*], Achilleas S. Frangakis[3*], Ruben C. Hartkoorn[2*], Marion Flipo[1*]

[#] Joint first authors: Anais Vieira Da Cruz, Juan-Carlos Jiménez-Castellanos, Clara Börnsen, Laurye Van Maele. These authors contributed equally.
[*] Joint last and corresponding authors: Klaas M. Pos, Achilleas S. Frangakis, Ruben Hartkoorn, Marion Flipo. These authors jointly supervised this work.
pos@em.uni-frankfurt.de
achilleas.frangakis@biophysik.org
ruben.hartkoorn@inserm.fr
marion.flipo@univ-lille.fr

Affiliations:
[1] Univ. Lille, Inserm, Institut Pasteur de Lille, U1177 - Drugs and Molecules for Living Systems, F-59000, Lille, France.
[2] Univ. Lille, CNRS, Inserm, CHU Lille, Institut Pasteur de Lille, U1019 - UMR 9017 - CIIL - Center for Infection and Immunity of Lille, F-59000 Lille, France.
[3] Buchmann Institute for Molecular Life Sciences and Institute for Biophysics, Goethe University Frankfurt, Max-von-Laue-Str. 15, D-60438 Frankfurt am Main, Germany.
[4] Institute of Biochemistry, Goethe-University Frankfurt, Max-von-Laue-Str. 9, D-60438 Frankfurt am Main, Germany.
[5] Univ. Lille, CNRS, Inserm, CHU Lille, Institut Pasteur Lille, US 41—UAR 2014—PLBS, F-59000 Lille, France

Table of content

Appendix Figure S1. Overview of cryo-EM processing of *K. pneumoniae* AcrB in complex with BDM91288……………………………………………………………………………………**2**
Appendix Table S1. Statistics of cryo-EM data collection and processing……………………**3**
Appendix Table S2. Refinement and validation statistics……………………………………**4**
Appendix Table S3. PK profile of BDM91288 (30 mg/kg) ……………………………………**5**
Appendix Table S4. PK profile of BDM91288 (30 mg/kg) in combination with levofloxacin (10 mg/kg).**6**
Appendix supplementary methods: Synthesis of PyrPip compounds **1'-13**……………………**7**
Appendix NMR spectra: NMR spectra of PyrPip compounds **1'-13**……………………………**19**

Appendix Figure S1. Overview of cryo-EM processing of *K. pneumoniae* AcrB in complex with BDM91288.

A Exemplary micrograph of the *Kp*AcrB sample with BDM91288 at -3.5 µm defocus with representative 2D class averages used for template picking.

B FSC curve of *Kp*AcrB.

C Top view and side view of *Kp*AcrB density map (C1 symmetry) with an overall resolution of 2.97 Å.

D Schematic processing overview of the *Kp*AcrB construct with bound BDM91288 inhibitor.

E map-model FSC curve.

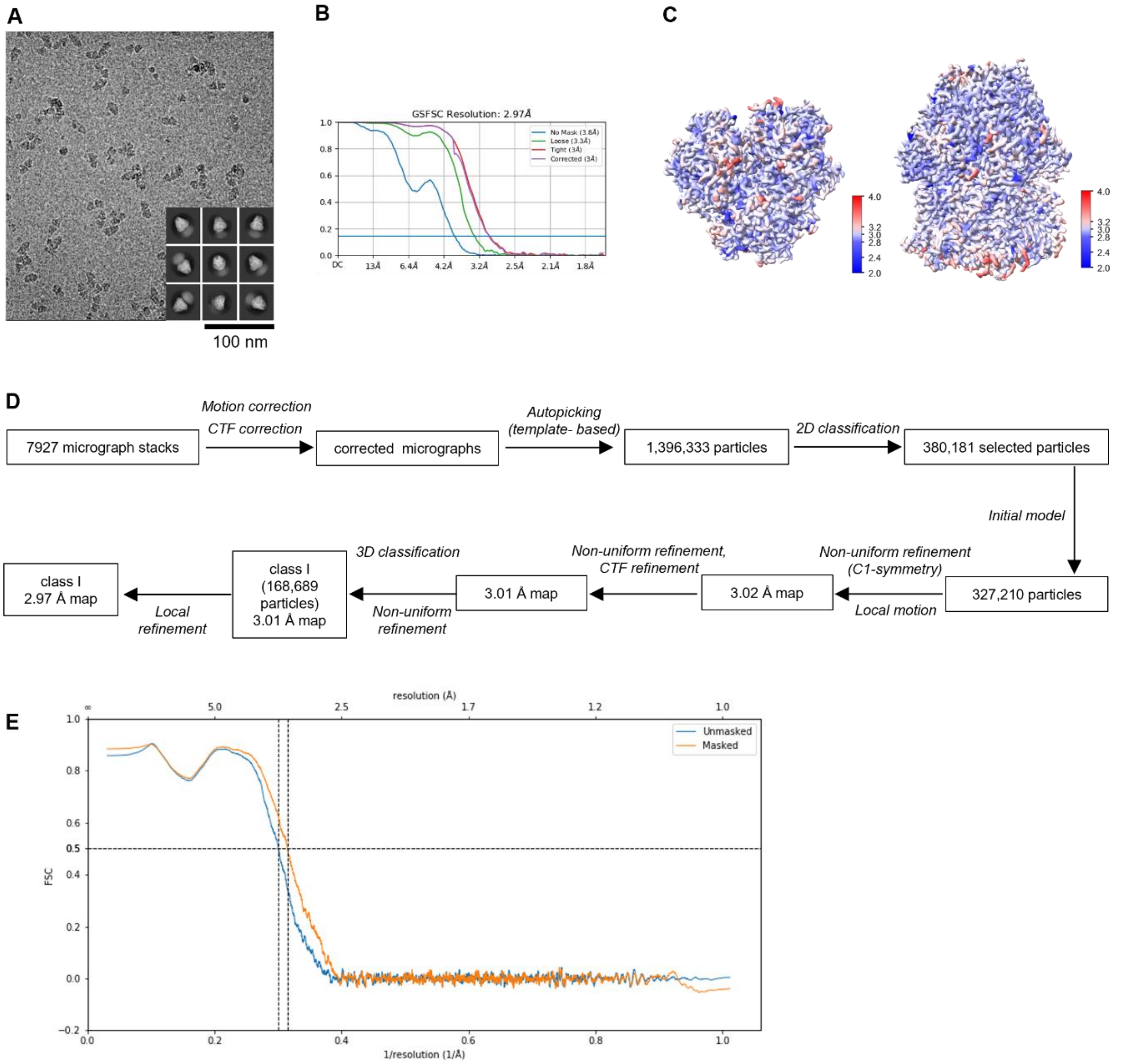

Appendix Table S1. Statistics of cryo-EM data collection and processing.

	*Kp*AcrB+BDM91288<br>EMDB**:** EMD-17350
Data collection & processing	
**Microscope**	Titan Krios G3i
**Detector**	Gatan K3
**Acquisition Software**	EPU 3.1
**Magnification**	105,000x
**Voltage (kV)**	300
**Electron exposure (e^-/Å^2)**	50
**Defocus range (μm)**	-0.8 to -3.5
**Pixel size (Å)**	0.837
**Symmetry imposed**	C1
**Initial particle images**	1,396,333
**Final particle images**	168,689
**Map Resolution (Å)**	2.97
**FSC threshold**	0.143
**Map resolution range (Å)**	2.0-4.0
**Number of frames**	50
**Micrographs used**	7927
**Processing software**	cryoSPARC v4.1.2
**Motion correction**	cryoSPARC v4.1.2
**CTF estimation**	cryoSPARC v4.1.2
**Particle images after 2D classification**	380,181
**Map sharpening B factor**	-120

Appendix Table S2. Refinement and validation statistics.

Refinement	***Kp*AcrB**<br>**PDB: 8P1I**
**Initial model used (PDB code)**	7OUK
**Model resolution (Å)**<br>**FSC threshold**	2.97<br>0.143
**Model resolution range (Å)**	2.0-4.0
**Map sharpening *B* factor ($Å^2$)**	-120
**Model composition**<br>**Atoms**<br>**Protein residues**<br>**Ligands**	 47459 (Hydrogens: 23948)<br>3077<br>4
**B-factors**<br>**Protein**<br>**Ligand**	 52.31<br>65.99
**R.m.s. deviations**<br>**Bond lengths (Å)**<br>**Bond angles (°)**	 0.004<br>0.514
**Validation**<br>**MolProbity score**<br>**Clashscore**<br>**Poor rotamers (%)**	 1.44<br>4.54<br>0.00
**Ramachandran plot**<br>**Favored (%)**<br>**Allowed (%)**<br>**Disallowed (%)**	 96.70<br>3.30<br>0.00

Appendix Table S3. PK profile of a single dose BDM91288 (5) (30 mg/kg, orally, formulated in 10% hydroxypropyl-β-cyclodextrin) (see Fig EV3A).

A. C_{max}, T_{max} and area under the curve in plasma and whole lung

BDM91288 (5)	Plasma	Whole lung
C_{max} (µM)	2.0	225.1
C_{max} (µg/g)		75
T_{max} (h)	4	4
$AUC_{(0\text{-}24h)}$ (h.µM)	27	3881
$AUC_{(0\text{-}24h)}$ (h. µg/g)		1288

B. Concentration values at 8 time points

BDM91288 (5)	Plasma		Whole lung		Whole lung	
Time (h)	$C_{average}$ (µM)	SD (µM)	$C_{average}$ (µM)	SD (µM)	$C_{average}$ (µg/g)	SD (µg/g)
0.25	0.8	0.2	21.3	6.7	7.1	2.2
0.5	0.8	0.4	35.4	23.7	11.7	7.9
1	1.6	0.3	93.5	6.4	31.0	2.1
1.5	1.3	1.0	101.9	49.7	33.8	16.5
2	1.8	0.1	159.9	11.5	53.1	3.8
4	2.0	0.5	225.1	17.8	74.7	5.9
6	1.4	0.8	168.3	18.3	55.9	6.1
24	0.5	0.4	159.1	23.7	52.8	7.9

Appendix Table S4. PK profile of a single dose BDM91288 (5) (30 mg/kg, orally, formulated in 10% hydroxypropyl-β-cyclodextrin) in combination with a single dose levofloxacin (10 mg/kg in water, i.p.) (see Fig EV3B).

A. Area under the curve in plasma, lung after bronchoalveolar lavage (BAL) and epithelial lining fluid (ELF)

**BDM91288 (5)**	**Plasma**	**Lung after BAL**	**ELF**
**$AUC_{(0\text{-}24h)}$ (h.µM)**	16	327	62

B. Concentration values at 2 h, 6 h and 24 h

**BDM91288 (5)**	**Plasma**		**Lung after BAL**		**Lung after BAL**		**ELF**	
**Time (h)**	**$C_{average}$ (µM)**	**SD (µM)**	**$C_{average}$ (µM)**	**SD (µM)**	**$C_{average}$ (µg/g)**	**SD (µg/g)**	**$C_{average}$ (µM)**	**SD (µM)**
**2**	1.03	0.23	52.7	13.8	**17.5**	4.6	**4.6**	1.1
**6**	0.77	0.03	73.2	7.2	**24.3**	2.4	**3.0**	0.6
**24**	0.55	0.10	75.4	4.5	**25.0**	1.5	**1.7**	0.2

Appendix supplementary methods: synthesis of PyrPip compounds 1'-13

General
Solvents for synthesis, analysis and purification were purchased as analytical grade from commercial suppliers and used directly without further purification. Chemical reagents were purchased from Fisher scientific, Fluorochem, Enamine or Sigma-Aldrich as reagent grade and used without further purification.

LC-MS Waters system was equipped with a 2747 sample manager, a 2695 separation module, a 2996 photodiode array detector (200-400 nm) and a Micromass ZQ2000 detector (scan 100-800). XBridge C18 column (50 mm x 4.6 mm, 3.5µm, Waters) was used. The injection volume was 20 µL. A mixture of water and acetonitrile was used as mobile phase in gradient-elution. The pH of the mobile phase was adjusted with HCOOH and NH_4OH to form a buffer solution at pH 3.8. The analysis time was 5 min (at a flow rate at 2 mL/min), 10 min (at a flow rate at 1 mL/min) or 30 min (at a flow rate at 1 mL/min). Purity (%) was determined by reversed phase HPLC, using UV detection (215 nm). All final compounds showed purity greater than 95%.

UPLC-MS Waters system was equipped with a UPLC I SMP MGR-FTN sample manager, an ACQUITY UPLC I-Class eK photodiode array detector (210-400 nm) and an ACQUITY QDa (Performance) detector (scan 30-1250). Acquity BEH C18 column (50 mm x 2.1 mm, 1.7 µm, Waters) was used. The injection volume was 0.5 µL. A mixture of water and acetonitrile was used as mobile phase in gradient-elution. The pH of the mobile phase was adjusted with HCOOH and NH_4OH to form a buffer solution at pH 3.8. The analysis time was 5 min (at a flow rate at 600 µL/min), 10 min (at a flow rate at 600 µL/min) or 30 min (at a flow rate at 600 µL/min). Purity (%) was determined using UV detection (215 nm), and all isolated compounds showed purity greater than 95%.

HRMS analysis was performed on a LC-MS system equipped with a LCT Premier XE mass spectrometer (Waters), using a XBridge C18 column (50 mm x 4.6 mm, 3.5µm, Waters). A gradient starting from 98% H_2O 5 mM Ammonium Formate pH 3.8 and reaching 100% MeCN 5 mM Ammonium Formate pH 3.8 within 3 min at a flow rate of 1 mL/min was used.

NMR spectra were recorded on a Bruker DRX-300 spectrometer. The results were calibrated to signals from the solvent as an internal reference [e.g. 2.50 (residual DMSO-d_6) and 39.52 (DMSO- d_6) ppm for ^1H and ^{13}C NMR spectra respectively]. Chemical shifts (δ) are in parts per million (ppm) downfield from tetramethylsilane (TMS). The assignments were made using one-dimensional (1D) ^1H and ^{13}C spectra and two-dimensional (2D) HSQC-DEPT, COSY and HMBC spectra. NMR coupling constants (J) are reported in Hertz (Hz), and splitting patterns are indicated as follows: s for singlet, brs for broad singlet, d for doublet, t for triplet, q for quartet, dd for doublet of doublet, ddd for doublet of doublet of doublet, m for multiplet, δ for chemical shift, *J* for coupling constant.

HRMS and NMR spectra for compound BDM91288 (**5**) are provided as Source data file.

Flash chromatography was performed using a Puriflash PF-430 with silica gel cartridges (Buchi silica 40 µm). ELSD and UV detection (254 nm) were used to collect the desired product. Reverse flash chromatography was performed using a CombiFlash® Rf200 with C_{18} cartridges (Buchi C_{18} 40 µm). UV detection (215 and 254 nm) was used to collect the desired product.

<u>Abbreviations</u>
Ac, acetate; Boc, tert-butoxycarbonyle; BINAP, 2,2'-bis(diphenylphosphino)-1,1'-binaphtyle; dba, dibenzylideneacetone; DMF, dimethylformamide; DCM, dichloromethane; DMSO, dimethylsulfoxide; Et, ethyl; EtOAc, ethyl acetate; MeCN, acetonitrile; MeOH, methanol; NCS, *N*-Chlorosuccinimide; ovn,

overnight; RT, room temperature; SEM, 2-(trimethylsilyl)ethoxymethyl; TBAF, tetra-*n*-butylammonium fluoride; *t*-Bu, tert-butyle; TEA, triethylamine; Xantphos, (9,9-Dimethyl-9*H*-xanthene-4,5-diyl)bis(diphenylphosphane).

__Procedure A:__ Introduction of piperazine on the quinoline by Buchwald reaction

The chlorinated derivative (0.39-8.7 mmol, 1.0 eq.), Boc-piperazine (1.5-3.3 eq.), *t*-BuONa (1.4-2.8 eq.), BINAP (0.02-0.08 eq.), $Pd(OAc)_2$ (0.02-0.04 eq.) were dissolved in toluene (0.6-11 mL) under argon. The mixture was heated at 110 °C overnight to 2 days, cooled to room temperature, dried under vacuum and purified by flash chromatography.

__Procedure B:__ Boc-cleavage with HCl

In a round-bottomed flask containing the corresponding Boc-protected compound (0.05-4.02 mmol, 1.0 eq.) in 1,4-dioxane (0.4-16 mL) was added HCl 4M in 1,4-dioxane (10-27 eq.). The mixture was stirred at room temperature during 1-2 days. The solvent was then evaporated under reduced pressure, petroleum ether was added and the mixture was filtered to give the desired compounds.

__Procedure C:__ Introduction of piperazine in position 3 of the quinoline by nucleophilic aromatic substitution

The chlorinated derivative (0.70-10.7 mmol, 1.0 eq.), Boc-piperazine (1.5-2.6 eq.) and triethylamine (1.3-2.6 eq.) were dissolved in acetonitrile (2.0-39.0 mL) under argon. The mixture was heated at 80 °C for 3 h to 2 days, cooled to room temperature. The reaction was washed with a 1M HCl aqueous solution, extracted twice with EtOAc. The organic layers were combined, washed with a saturated solution of NaCl, dried over $MgSO_4$, evaporated under vacuum and purified by flash chromatography.

__Procedure D:__ Introduction of amine in position 6 of the quinoline by Buchwald reaction

tert-butyl 4-(6-bromo-3-chloro-2-quinolyl)piperazine-1-carboxylate or *tert*-butyl 4-(3-chloro-6-iodo-2-quinolyl)piperazine-1-carboxylate (0.17-0.72 mmol, 1.0 eq.), corresponding amine (1.0-3.2 eq.), Cs_2CO_3 (1.3-2.8 eq.), Xantphos (0.08-0.12 eq.), Pd_2dba_3 (0.02-0.07 eq.) were dissolved in dioxane (0.8-4.0 mL) under argon. The mixture was heated at 100 °C overnight to 2 days, cooled to room temperature, dried under vacuum and purified by flash chromatography.

- __Synthesis of compound **1'**:__

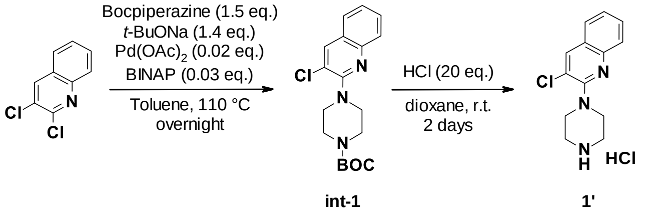

int-1 **1'**

***tert*-butyl 4-(3-chloro-2-quinolyl)piperazine-1-carboxylate (int-1):**
The **int-1** was obtained using **procedure A**, from 2,3-dichloroquinoline (8.7 mmol, 1.0 eq.), Boc-piperazine (1.5 eq.), *t*-BuONa (1.4 eq.), BINAP (0.03 eq.), $Pd(OAc)_2$ (0.02 eq.) in toluene (11 mL) at 110 °C overnight. The crude was purified with a gradient of cyclohexane/EtOAc 100:0 to 80:20 to give **int-1** as a yellow oil.

Yield: 48%; **^{1}H NMR (300 MHz, CD_2Cl_2):** δ 1.47 (s, 9H), 3.40-3.45 (m, 4H), 3.59-3.63 (m, 4H), 7.40 (ddd, *J* = 1.2, 7.0, 8.1 Hz, 1H), 7.62 (ddd, *J* = 1.5, 7.0, 8.5 Hz, 1H), 7.65-7.68 (m, 1H), 7.79-7.83 (m, 1H), 8.08 (s, 1H) ppm; **LCMS (ES+)** *m/z* $[M+H]^+$ 348.

3-chloro-2-piperazin-1-yl-quinoline;hydrochloride (1'):
The **int-1** (4.02 mmol, 1.0 eq.) was deprotected using **procedure B** with HCl 4M (20 eq.) in 1,4-dioxane (16 mL) for 2 days to give **1'** as a white powder.
Yield: 100%; **Purity (215 nm)** > 99%; **^{1}H NMR (300 MHz, DMSO-*d*$_6$):** δ 3.24-3.28 (m, 4H), 3.63-3.68 (m, 4H), 7.49 (ddd, *J* = 1.1, 7.0, 8.0 Hz, 1H), 7.70 (ddd, *J* = 1.4, 7.0, 8.4 Hz, 1H), 7.82 (d, *J* = 8.4 Hz, 1H), 7.87 (dd, *J* = 1.0, 7.4 Hz, 1H), 8.50 (s, 1H), 9.62 (br s, 2H) ppm; **^{13}C NMR (75 MHz, DMSO-*d*$_6$):** δ 42.5, 45.9, 121.5, 125.6, 125.7, 126.9, 127.0, 130.3, 138.3, 144.5, 155.5 ppm; **LCMS (ES+)** *m/z* $[M+H]^+$ 248; **HRMS (*m/z*)**: $[M+H]^+$ calcd. for $C_{13}H_{15}ClN_3$ 248.0955; found 248.0958.

- Synthesis of compound **2**:

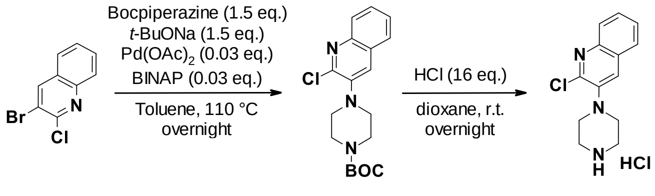

***tert*-butyl 4-(2-chloro-3-quinolyl)piperazine-1-carboxylate (int-2):**
The **int-2** was obtained using **procedure A**, from 3-bromo-2-chloro-quinoline (0.78 mmol, 1.0 eq.), Boc-piperazine (1.5 eq.), *t*-BuONa (1.5 eq.), BINAP (0.03 eq.), $Pd(OAc)_2$ (0.03 eq.) in toluene (1.7 mL) at 110 °C overnight. The crude was purified with a gradient of cyclohexane/EtOAc 100:0 to 90:10 to give **int-2** as a yellow oil.
Yield: 6%; **^{1}H NMR (300 MHz, CD_2Cl_2):** δ 1.48 (s, 9H), 3.09 (t, *J* = 4.9 Hz, 4H), 3.64 (t, *J* = 4.9 Hz, 4H), 7.53 (ddd, *J* = 1.4, 7.0, 8.1 Hz, 1H), 7.59-7.65 (m, 2H), 7.77 (dd, *J* = 1.0, 7.0 Hz, 1H), 7.91 (td, *J* = 0.6, 8.3 Hz, 1H) ppm; **LCMS (ES+)** *m/z* $[M+H]^+$ 348.

2-chloro-3-piperazin-1-yl-quinoline;hydrochloride (2):
The **int-2** (0.05 mmol, 1.0 eq.) was deprotected using **procedure B** with HCl 4M (16 eq.) in 1,4-dioxane (0.4 mL) overnight to give **2** as a yellow solid.
Yield: 100%; **Purity (215 nm)** > 99%; **^{1}H NMR (300 MHz, DMSO-*d*$_6$):** δ 3.29-3.47 (m, 8H), 7.60-7.74 (m, 2H), 7.90 (d, *J* = 7.7 Hz, 1H), 8.00 (d, *J* = 7.1 Hz, 1H), 8.14 (s, 1H), 9.44 (br s, 2H) ppm; **^{13}C NMR (75 MHz, DMSO-*d*$_6$):** δ 42.9, 48.0, 126.6, 127.3, 127.4, 127.5, 127.9, 129.1, 142.0, 143.2, 146.9 ppm; **LCMS (ES+)** *m/z* $[M+H]^+$ 248; **HRMS (*m/z*):** $[M+H]^+$ calcd. for $C_{13}H_{15}ClN_3$ 248.0955; found 248.0948.

- Synthesis of compound **3**:

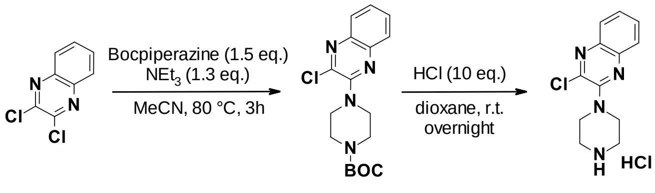

***tert*-butyl 4-(3-chloroquinoxalin-2-yl)piperazine-1-carboxylate (int-3):**
The **int-3** was obtained using **procedure C**, from 2,3-dichloroquinoxaline (0.70 mmol, 1.0 eq.), Boc-piperazine (1.5 eq.), NEt_3 (1.3 eq.) in MeCN (2.0 mL) at 80 °C for 3 h to give **int-3** as an orange oil.
Yield: 100%; **^{1}H NMR (300 MHz, CD_2Cl_2):** δ 1.46 (s, 9H), 3.46-3.52 (m, 4H), 3.59-3.64 (m, 4H), 7.53-7.59 (m, 1H), 7.64-7.69 (m, 1H), 7.81-7.88 (m, 2H) ppm; **LCMS (ES+)** *m/z* $[M+H]^+$ 349.

2-chloro-3-piperazin-1-yl-quinoxaline;hydrochloride (3):
The **int-3** (0.70 mmol, 1.0 eq.) was deprotected using **procedure B** with HCl 4M (10 eq.) in 1,4-dioxane (1.7 mL) overnight. The crude product was purified with a gradient of MeOH/H_2O 90:10 to 00:100 to give **3** as a white powder.
Yield: 42%; **Purity (215 nm)**: 99%; **^{1}H NMR (300 MHz, DMSO-d_6):** δ 3.26-3.29 (m, 4H), 3.73-3.76 (m, 4H), 7.67 (ddd, *J* = 1.5, 6.9, 8.2 Hz, 1H), 7.78 (ddd, *J* = 1.5, 6.9, 8.5 Hz, 1H), 7.85-7.93 (m, 2H), 9.65 (br s, 2H) ppm; **^{13}C NMR (75 MHz, DMSO-d_6):** δ 42.2, 45.5, 126.9, 127.4, 128.1, 130.7, 137.9, 139.2, 141.2, 151.7 ppm; **LCMS (ES+)** *m/z* $[M+H]^+$ 249; **HRMS (*m/z*)**: $[M+H]^+$ calcd. for $C_{12}H_{14}ClN_4$ 249.0907; found 249.0913.

- Synthesis of compound **4**:

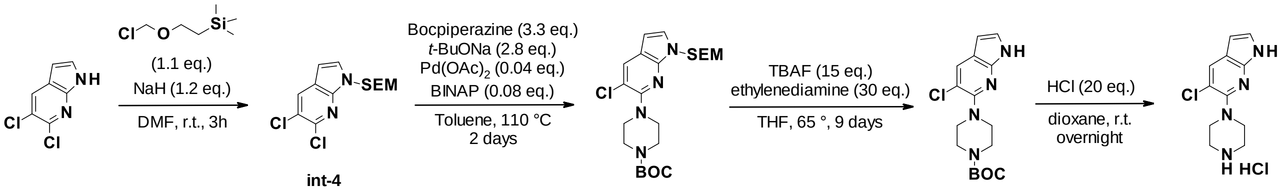

***tert*-butyl 4-[5-chloro-1-(2-trimethylsilylethoxymethyl)pyrrolo[2,3-b]pyridin-6-yl]piperazine-1-carboxylate (int-5) :**
From 5,6-dichloro-1*H*-pyrrolo[2,3-b]pyridine (0.39 mmol, 1.0 eq.) in DMF (0.6 mL) was added NaH (1.2 eq.) at 0 °C then (trimethylsilyl)ethoxymethyl chloride (1.1 eq.) was added dropwise and the reaction was stirred at room temperature for 3 h. The reaction was quenched with water, extracted thrice with DCM. The organic layers were combined, washed with a saturated solution of NaCl, dried over $MgSO_4$, evaporated under vacuum to give **int-4** as a colorless oil.

The crude **int-4** (0.25 mmol, 1.0 eq.) was reacted using **procedure A** with Boc-piperazine (3.3 eq), *t*-BuONa (2.8 eq), BINAP (0.08 eq), $Pd(OAc)_2$ (0.04 eq) in toluene (0.6 mL) at 110 °C overnight. The crude was purified with a gradient of cyclohexane/EtOAc 100:0 to 70:30 to give **int-5** as a yellow solid.
Yield: 40% in 2 steps; **^{1}H NMR (300 MHz, CD_2Cl_2):** δ 0.07 (s, 9H), 0.86-0.92 (m, 2H), 1.47 (s, 9H), 3.24-3.29 (m, 4H), 3.52-3.61 (m, 6H), 5.55 (s, 2H), 6.39 (d, *J* = 3.6 Hz, 1H), 7.22 (d, *J* = 3.6 Hz, 1H), 7.85 (s, 1H) ppm; **LCMS (ES+)** *m/z* $[M+H]^+$ 467.

***tert*-butyl 4-(5-chloro-1*H*-pyrrolo[2,3-b]pyridin-6-yl)piperazine-1-carboxylate (int-6):**
From **int-5** (0.14 mmol, 1.0 eq.) in THF (1.2 mL) was added TBAF (3.0 eq.) and ethylenediamine (6.0 eq.) and the reaction was stirred at 65 °C. Small portions of TBAF (3.0 eq.) and ethylenediamine (6.0 eq.) were added 4 times during 9 days. The reaction was quenched with water, extracted thrice with EtOAc. The organic layers were combined, washed with a saturated solution of NaCl, dried over $MgSO_4$, evaporated under vacuum. The crude was purified with a gradient of cyclohexane/EtOAc 100:0 to 80:20 to give **int-6** as a colorless oil.
Yield: 81%; **^{1}H NMR (300 MHz, CD_2Cl_2):** δ 1.47 (s, 9H), 3.21-3.25 (m, 4H), 3.58-3.62 (m, 4H), 6.38 (dd, *J* = 2.0, 3.5 Hz, 1H), 7.19 (d, *J* = 2.4, 3.5 Hz, 1H), 7.88 (d, *J* = 0.5 Hz, 1H), 9.02 (br s, 1H) ppm; **LCMS (ES+)** *m/z* $[M+H]^+$ 337.

5-chloro-6-piperazin-1-yl-1*H*-pyrrolo[2,3-b]pyridine;hydrochloride (4):
The **int-6** (0.11 mmol, 1.0 eq.) was deprotected using **procedure B** with HCl 4M (20 eq.) in 1,4-dioxane (0.7 mL) overnight, to give **4** as a yellow solid.
Yield: 100%; **Purity (215 nm)**: 99%; **^{1}H NMR (300 MHz, DMSO-d_6):** δ 3.21-3.26 (m, 4H), 3.38-3.42 (m, 4H), 6.34-6.37 (m, 1H), 7.36-7.38 (m, 1H), 8.04 (s, 1H), 9.38 (br s, 2H), 11.69 (br s, 1H) ppm; **^{13}C NMR (75

MHz, DMSO-d_6): δ 42.8, 46.6, 99.6, 115.1, 116.8, 126.0, 130.7, 145.0, 151.8 ppm; **LCMS (ES+)** *m/z* $[M+H]^+$ 237; **HRMS (*m/z*)**: $[M+H]^+$ calcd. for $C_{11}H_{14}ClN_4$ 237.0907; found 237.0898.

- Synthesis of compounds **5-7**:

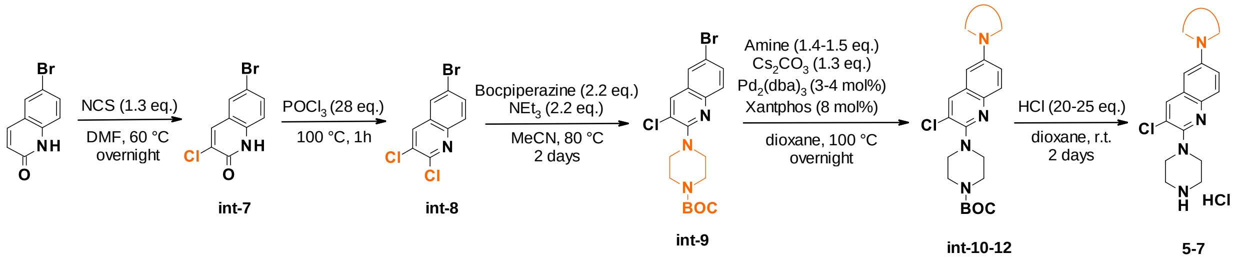

6-bromo-3-chloro-1*H*-quinolin-2-one (int-7):
To a solution of 6-bromo-1*H*-quinolin-2-one (22.3 mmol, 1.0 eq.) in anhydrous DMF (50.0 mL) was added NCS (1.3 eq.) and the reaction was stirred at 60 °C overnight. The DMF was evaporated and the compound was rinsed with water, EtOAc and MeOH and filtered under vacuum to give **int-7** as a pink solid.
Yield: 73%; **^{1}H NMR (300 MHz, CD_2Cl_2):** δ 7.28 (d, *J* = 8.8 Hz, 1H), 7.67 (dd, *J* = 2.3, 8.8 Hz, 1H), 7.92 (d, *J* = 2.2 Hz, 1H), 8.27 (s, 1H), 12.42 (s, 1H) ppm; **LCMS (ES+)** *m/z* $[M+H]^+$ 258.

6-bromo-2,3-dichloro-quinoline (int-8):
To a solution of 6-bromo-3-chloro-1*H*-quinolin-2-one **int-7** (16.3 mmol, 1.0 eq.) was added $POCl_3$ (28 eq.). The mixture was stirred at 100 °C for 1 h, then the reaction mixture was poured on ice, basified with a saturated solution of Na_2CO_3 until pH = 7. The aqueous solution was extracted thrice with EtOAc. The organic layer was washed with brine, dried over $MgSO_4$ and then evaporated under reduced pressure to give **int-8** as a brown solid.
Yield: 73%; **^{1}H NMR (300 MHz, CD_2Cl_2):** δ 7.83 (dd, *J* = 2.0, 9.0 Hz, 1H), 7.88 (d, *J* = 9.2 Hz, 1H), 7.98 (d, *J* = 2.0 Hz, 1H), 8.21 (s, 1H) ppm; **LCMS (ES+)** *m/z* $[M+H]^+$ 276.

***tert*-butyl 4-(6-bromo-3-chloro-2-quinolyl)piperazine-1-carboxylate (int-9):**
The **int-9** was obtained using **procedure C**, from 6-bromo-2,3-dichloro-quinoline **int-8** (10.7 mmol, 1.0 eq.), Boc-piperazine (2.2 eq.), NEt_3 (2.2 eq.) in MeCN (39.0 mL) at 80 °C for 2 days. The crude was purified with a gradient of cyclohexane/EtOAc 100:0 to 90:10 to give **int-9** as a pale-yellow solid.
Yield: 85%; **^{1}H NMR (300 MHz, CD_2Cl_2):** δ 1.47 (s, 9H), 3.41-3.45 (m, 4H), 3.58-3.62 (m, 4H), 7.67-7.68 (m, 2H), 7.80-7.81 (m, 1H), 7.99 (s, 1H) ppm; **LCMS (ES+)** *m/z* $[M+H]^+$ 426.

***tert*-butyl 4-[2-(4-tert-butoxycarbonylpiperazin-1-yl)-3-chloro-6-quinolyl]piperazine-1-carboxylate (int-10):**

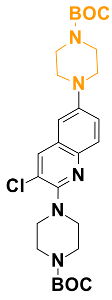

int-10

The **int-10** was obtained using **procedure D**, from *tert*-butyl 4-(6-bromo-3-chloro-2-quinolyl)piperazine-1-carboxylate **int-9** (0.72 mmol, 1.0 eq.), Boc-piperazine (1.5 eq.), Cs_2CO_3 (1.3 eq.), Xantphos (0.08 eq.), Pd_2dba_3 (0.04 eq.) in dioxane (4.0 mL) at 100 °C overnight. The crude product was purified with a gradient of cyclohexane/EtOAc 100:0 to 40:60 to give **int-10** as a pale-yellow solid.
Yield: 48%; **^{1}H NMR (300 MHz, CD_2Cl_2):** δ 1.46 (s, 18H), 3.18-3.21 (m, 4H), 3.32-3.35 (m, 4H), 3.58-3.61 (m, 8H), 6.93 (d, *J* = 2.6 Hz, 1H), 7.40 (dd, *J* = 2.6, 9.2 Hz, 1H), 7.71 (d, *J* = 9.3 Hz, 1H), 7.95 (s, 1H) ppm; **LCMS (ES+)** *m/z* $[M+H]^+$ 532.

3-chloro-2,6-di(piperazin-1-yl)quinoline;dihydrochloride (5):

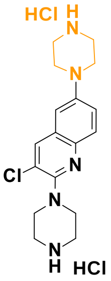

5

The **int-10** (0.34 mmol, 1.0 eq.) was deprotected using **procedure B** with HCl 4M (25 eq.) in 1,4-dioxane (2.8 mL) for 2 days to give **5** as an orange solid.
Yield: 100%; **Purity (215 nm)** : 98%; **^{1}H NMR (300 MHz, DMSO-*d*$_6$):** δ 3.22-3.27 (m, 8H), 3.47-3.57 (m, 8H), 7.24 (d, *J* = 2.2 Hz, 1H), 7.57 (dd, *J* = 2.4, 9.3 Hz, 1H), 7.72 (d, *J* = 9.2 Hz, 1H), 8.30 (s, 1H), 9.38 (br s, 4H) ppm; **^{13}C NMR (75 MHz, DMSO-*d*$_6$):** δ 42.5, 42.6, 45.6, 46.1, 109.0, 122.0, 122.8, 126.8, 128.0, 136.8, 139.9, 147.5, 153.8 ppm; **LCMS (ES+)** *m/z* [M+H]$^+$ 332; **HRMS (*m/z*)**: [M+H]$^+$ calcd. for $C_{17}H_{23}ClN_5$ 332.1642; found 332.1636.

***tert*-butyl 4-(3-chloro-6-morpholino-2-quinolyl)piperazine-1-carboxylate (int-11):**

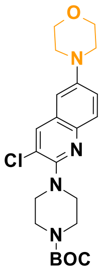

int-11

The **int-11** was obtained using **procedure D**, from *tert*-butyl 4-(6-bromo-3-chloro-2-quinolyl)piperazine-1-carboxylate **int-9** (0.35 mmol, 1.0 eq), morpholine (1.5 eq.), Cs_2CO_3 (1.3 eq.), Xantphos (0.08 eq.), Pd_2dba_3 (0.04 eq.) in dioxane (2.0 mL) at 100 °C overnight. The crude product was purified with a gradient of cyclohexane/EtOAc 100:0 to 85:15 to give **int-11** as a yellow solid.
Yield: 80%; **^{1}H NMR (300 MHz, CD_2Cl_2):** δ 1.47 (s, 9H), 3.19-3.23 (m, 4H), 3.31-3.36 (m, 4H), 3.58-3.62 (m, 4H), 3.84-3.88 (m, 4H), 6.91 (d, *J* = 2.7 Hz, 1H), 7.38 (dd, *J* = 2.7, 9.3 Hz, 1H), 7.71 (d, *J* = 9.2 Hz, 1H), 7.95 (s, 1H) ppm; **LCMS (ES+)** *m/z* [M+H]$^+$ 433.

4-(3-chloro-2-piperazin-1-yl-6-quinolyl)morpholine;hydrochloride (6):

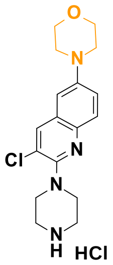

6

The **int-11** (0.28 mmol) was deprotected using **procedure B** with HCl 4M (20 eq.) in 1,4-dioxane (2.5 mL) for 2 days to give **6** as a yellow solid.
Yield: 89%; **Purity (215 nm)**: 99%; **^{1}H NMR (300 MHz, DMSO-*d*$_6$):** δ 3.22-3.27 (m, 8H), 3.53-3.57 (m, 4H), 3.78-3.83 (m, 4H), 7.24 (d, *J* = 2.2 Hz, 1H), 7.60 (dd, *J* = 2.4, 9.3 Hz, 1H), 7.72 (d, *J* = 9.2 Hz, 1H), 8.30 (s, 1H), 9.48 (s, 2H) ppm; **^{13}C NMR (75 MHz, DMSO-*d*$_6$):** δ 42.5, 46.1, 48.9, 65.8, 108.6, 122.0, 122.1, 126.8, 127.7, 136.9, 139.7, 147.9, 153.6 ppm; **LCMS (ES+)** *m/z* [M+H]$^+$ 333; **HRMS (*m/z*)**: [M+H]$^+$ calcd. for $C_{17}H_{22}ClN_4O$ 333.1482; found 333.1491.

***tert*-butyl 4-[3-chloro-6-(1-piperidyl)-2-quinolyl]piperazine-1-carboxylate (int-12):**

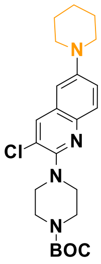

int-12

The **int-12** was obtained using **procedure D**, from *tert*-butyl 4-(6-bromo-3-chloro-2-quinolyl)piperazine-1-carboxylate **int-9** (0.35 mmol, 1.0 eq), piperidine (1.4 eq.), Cs_2CO_3 (1.3 eq.), Xantphos (0.08 eq.), Pd_2dba_3 (0.03 eq.) in dioxane (2.0 mL) at 100 °C overnight. The crude product was purified with a gradient of cyclohexane/EtOAc 100:0 to 85:15 to give **int-12** as a yellow oil.
Yield: 83%; **^{1}H NMR (300 MHz, CD_2Cl_2):** δ 1.47 (s, 9H), 1.60-1.65 (m, 2H), 1.64-1.78 (m, 4H), 3.20-3.24 (m, 4H), 3.30-3.34 (m, 4H), 3.58-3.62 (m, 4H), 6.90 (d, *J* = 2.7 Hz, 1H), 7.39 (dd, *J* = 2.8, 9.3 Hz, 1H), 7.67 (d, *J* = 9.3 Hz, 1H), 7.92 (s, 1H) ppm; **LCMS (ES+)** *m/z* [M+H]$^+$ 431.

3-chloro-2-piperazin-1-yl-6-(1-piperidyl)quinoline;hydrochloride (7):

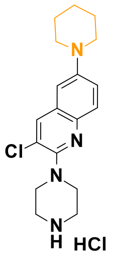

7

The **int-12** (0.30 mmol, 1.0 eq.) was deprotected using **procedure B** with HCl 4M (20 eq.) in 1,4-dioxane (2.5 mL) for 2 days to give **7** as a yellow solid.
Yield: 97%; **Purity (215 nm)**: 99%; **^{1}H NMR (300 MHz, DMSO-d_6):** δ 1.64-1.72 (m, 2H), 1.93-2.01 (m, 4H), 3.25-3.29 (m, 4H), 3.49-3.53 (m, 4H), 3.62-3.67 (m, 4H), 7.89 (d, *J* = 9.2 Hz, 1H), 8.02-8.16 (m, 2H), 8.47 (s, 1H), 9.53 (s, 2H) ppm; **^{13}C NMR (75 MHz, DMSO-d_6):** δ 21.6, 23.4, 42.4, 45.8, 54.4, 116.7, 122.6, 123.5, 125.6, 125.6, 128.6, 138.0, 142.7, 155.6 ppm; **LCMS (ES+)** *m/z* [M+H]$^+$ 331; **HRMS (*m/z*)**: [M+H]$^+$ calcd. for $C_{18}H_{24}ClN_4$ 331.1689; found 331.1707.

- Synthesis of compound **8:**

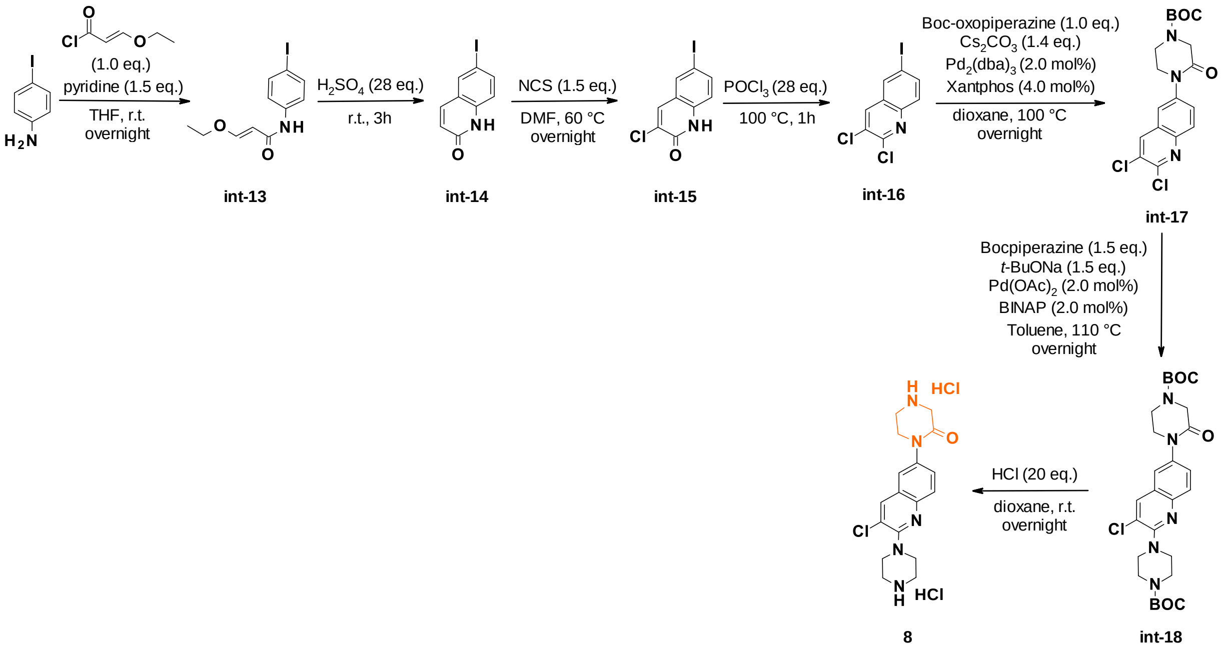

(*E*)-3-ethoxy-*N*-(4-iodophenyl)prop-2-enamide (int-13):
To a solution of 3-ethoxyacryloyl chloride (1.0 eq.) in THF (9.2 mL) at 0 °C, was added 4-iodoaniline (9.13 mmol, 1.0 eq.) and pyridine (1.5 eq.). The mixture was stirred overnight at room temperature. The reaction was quenched with water, extracted thrice with EtOAc, washed with brine, dried over $MgSO_4$, and then evaporated under reduced pressure. The crude product was purified with a gradient of cyclohexane/EtOAc 100:0 to 60:40 to give **int-13** as a pale-yellow solid.
Yield: 88%; **^{1}H NMR (300 MHz, CD_2Cl_2):** δ 1.27 (t, *J* = 7.0 Hz, 3H), 3.95 (q, *J* = 7.0 Hz, 2H), 5.49 (d, *J* = 12.3 Hz, 1H), 7.43-7.51 (m, 3H), 7.61 (d, *J* = 8.8 Hz, 2H), 9.82 (s, 1H) ppm; **LCMS (ES+)** *m/z* [M+H]$^+$ 318.

6-iodo-1*H*-quinolin-2-one (int-14):
(*E*)-3-ethoxy-*N*-(4-iodophenyl)prop-2-enamide **int-13** (7.1 mmol, 1.0 eq) was added by small portion at 0 °C to concentrated sulfuric acid (28 eq.). The resulting mixture was stirred at room temperature for 3h. The suspension was cooled at 0 °C and quenched with a solution of NaOH until pH = 7-8, extracted thrice with EtOAc, washed with brine, dried over $MgSO_4$ and then evaporated under reduced pressure to afford to **int-14** as a pale-yellow solid.
Yield: 74%; **^{1}H NMR (300 MHz, CD_2Cl_2):** δ 6.51 (d, *J* = 9.6 Hz, 1H), 7.11 (d, *J* = 8.6 Hz, 1H), 7.76 (dd, *J* = 2.0, 8.6 Hz, 1H), 7.84 (d, *J* = 9.6 Hz, 1H), 8.06 (d, *J* = 1.9 Hz, 1H), 11.83 (br s, 1H) ppm; **LCMS (ES+)** *m/z* [M+H]$^+$ 272.

3-chloro-6-iodo-1*H*-quinolin-2-one (int-15):
To a solution of 6-iodo-1*H*-quinolin-2-one **int-14** (3.7 mmol, 1.0 eq.) in anhydrous DMF (10.0 mL) was added NCS (1.5 eq.) and the reaction was stirred at 60 °C overnight. The DMF was evaporated and the compound was quenched with water, extracted thrice with EtOAc. The organic layer was washed with brine, dried over $MgSO_4$ and then evaporated under reduced pressure. The crude product was purified by flash chromatography (cyclohexane/EtOAc 100/0-70/30) to give **int-15** as a pale-yellow solid.
Yield: 91%; **^{1}H NMR (300 MHz, CD_2Cl_2):** δ 7.14 (d, *J* = 8.7 Hz, 1H), 7.81 (dd, *J* = 2.0, 8.6 Hz, 1H), 8.08 (d, *J* = 2.0 Hz, 1H), 8.25 (s, 1H), 12.38 (s, 1H) ppm; **LCMS (ES+)** *m/z* $[M+H]^+$ 306.

2,3-dichloro-6-iodo-quinoline (int-16):
To a solution of 3-chloro-6-iodo-1*H*-quinolin-2-one **int-15** (2.0 mmol, 1.0 eq.) was added $POCl_3$ (28 eq.). The mixture was stirred at 100°C for 1 h, then reaction mixture was poured on ice, basified with a saturated solution of Na_2CO_3 until pH = 7. The aqueous solution was extracted thrice with EtOAc. The organic layer was washed with brine, dried over $MgSO_4$ and then evaporated under reduced pressure to give **int-16** as a green solid.
Yield: 85%; **^{1}H NMR (300 MHz, CD_2Cl_2):** δ 7.76 (d, *J* = 8.9 Hz, 1H), 8.10 (dd, *J* = 2.0, 8.9 Hz, 1H), 8.50 (d, *J* = 2.0 Hz, 1H), 8.73 (s, 1H) ppm.

***tert*-butyl 4-(2,3-dichloro-6-quinolyl)-3-oxo-piperazine-1-carboxylate (int-17):**
The **int-17** was obtained using **procedure D**, 2,3-dichloro-6-iodo-quinoline **int-16** (0.6 mmol, 1.0 eq.), *tert*-butyl 3-oxopiperazine-1-carboxylate (1.0 eq.), Cs_2CO_3 (1.4 eq.), Xantphos (0.04 eq.), Pd_2dba_3 (0.02 eq.) were dissolved in 1,4-dioxane (2.5 mL) at 100 °C overnight. The crude product was purified by flash chromatography (cyclohexane/EtOAc 100/0-50/50) to give **int-17** as a white solid. **Yield**: 84%; **^{1}H NMR (300 MHz, CD_2Cl_2):** δ 1.49 (s, 9H), 3.81-3.84 (m, 4H), 4.25 (s, 2H), 7.70 (d, *J* = 2.3 Hz, 1H), 7.75 (dd, *J* = 2.4, 9.0 Hz, 1H), 8.00 (d, *J* = 9.0 Hz, 1H), 8.26 (s, 1H) ppm; **LCMS (ES+)** *m/z* $[M+H]^+$ 396.

***tert*-butyl 4-[2-(4-tert-butoxycarbonylpiperazin-1-yl)-3-chloro-6-quinolyl]-3-oxo-piperazine-1-carboxylate (int-18):**
The **int-18** was obtained using **procedure A**, from *tert*-butyl 4-(2,3-dichloro-6-quinolyl)-3-oxo-piperazine-1-carboxylate **int-17** (0.4 mmol, 1.0 eq), *tert*-butyl piperazine-1-carboxylate (1.5 eq.), *t*-BuONa (1.4 eq.), BINAP (0.02 eq.), $Pd(OAc)_2$ (0.02 eq.) in toluene (1.8 mL) at 110 °C overnight. The crude product was purified by flash chromatography (cyclohexane/EtOAc 100/0-20/80) to give **int-18** as a yellow solid.
Yield: 26%; **^{1}H NMR (300 MHz, CD_2Cl_2):** δ 1.47 (s, 9H), 1.49 (s, 9H), 3.41-3.46 (m, 4H), 3.59-3.63 (m, 4H), 3.79 (s, 4H), 4.22 (s, 2H), 7.54-7.58 (m, 2H), 7.82 (dd, *J* = 0.7, 9.6 Hz, 1H), 8.05 (s, 1H) ppm; **LCMS (ES+)** *m/z* $[M+H]^+$ 546.

1-(3-chloro-2-piperazin-1-yl-6-quinolyl)piperazin-2-one;dihydrochloride (8):
The **int-18** (0.12 mmol, 1.0 eq.) was deprotected using **procedure B** with HCl 4M (20 eq.) in 1,4-dioxane (1.0 mL) overnight. The crude product was purified by reverse phase chromatography ($MeOH/H_2O$ 10/90-100/0) to give **8** as a yellow powder.
Yield: 74%; **Purity (215 nm)**: 99%; **^{1}H NMR (300 MHz, DMSO-*d*$_6$):** δ 3.24-3.29 (m, 4H), 3.55-3.66 (m, 6H), 3.87-3.90 (m, 2H), 3.98-4.02 (m, 2H), 7.68 (dd, *J* = 1.3, 9.0 Hz, 1H), 7.81 (d, *J* = 1.3 Hz, 1H), 7.84 (d, *J* = 9.0 Hz, 1H), 8.53 (s, 1H), 9.57 (br s, 2H), 10.24 (br s, 2H) ppm; **^{13}C NMR (75 MHz, DMSO-*d*$_6$):** δ 39.6, 42.4, 45.0, 45.8, 46.2, 122.0, 122.7, 125.6, 127.7, 128.7, 138.0, 138.4, 143.1, 155.7, 162.2 ppm; **LCMS (ES+)** *m/z* $[M+H]^+$ 346; **HRMS (*m/z*)**: $[M+H]^+$ calcd. for $C_{17}H_{21}ClN_5O$ 346.1435; found 346.1434.

- <u>Synthesis of compounds **9-11**:</u>

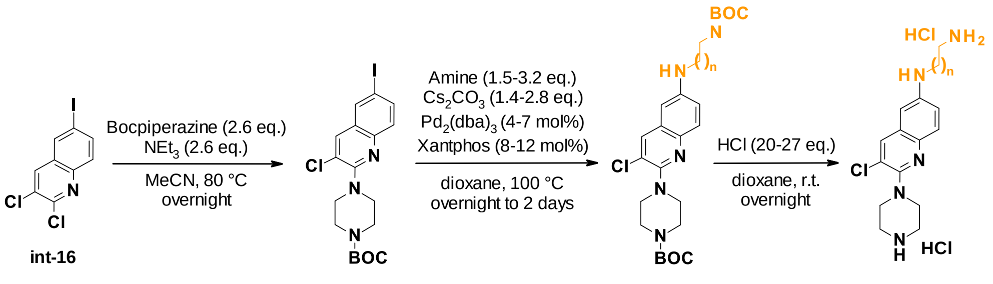

***tert*-butyl 4-(3-chloro-6-iodo-2-quinolyl)piperazine-1-carboxylate (int-19):**

The **int-19** was obtained using **procedure C**, from 2,3-dichloro-6-iodo-quinoline **int-16** (1.08 mmol, 1.0 eq.), Boc-piperazine (2.6 eq.), NEt_3 (2.6 eq.) in MeCN (3.9 mL) at 80 °C overnight. The crude was purified with a gradient of cyclohexane/EtOAc 100:0 to 40:60 to give **int-19** as a pale-yellow solid.
Yield: 80%; **^{1}H NMR (300 MHz, CD_2Cl_2):** δ 1.47 (s, 9H), 3.41-3.45 (m, 4H), 3.58-3.62 (m, 4H), 7.54 (d, *J* = 8.8 Hz, 1H), 7.84 (dd, *J* = 2.0, 8.8 Hz, 1H), 7.94 (s, 1H), 8.00 (d, *J* = 2.0 Hz, 1H) ppm; **LCMS (ES+)** *m/z* $[M+H]^+$ 474.

***tert*-butyl 4-[6-[2-(*tert*-butoxycarbonylamino)ethylamino]-3-chloro-2-quinolyl]piperazine-1-carboxylate (int-20):**

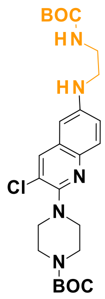

int-20

The **int-20** was obtained using **procedure D**, from *tert*-butyl 4-(3-chloro-6-iodo-2-quinolyl)piperazine-1-carboxylate **int-19** (0.17 mmol, 1.0 eq.), *tert*-butyl *N*-(2-aminoethyl)carbamate (3.2 eq.), Cs_2CO_3 (2.8 eq.), Xantphos (0.12 eq.), Pd_2dba_3 (0.07 eq.) in dioxane (0.8 mL) at 100 °C for 2 days. The crude product was purified with a gradient of cyclohexane/EtOAc 100:0 to 70:30 to give **int-20** as a yellow solid.
Yield: 43%; **^{1}H NMR (300 MHz, CD_2Cl_2):** δ 1.44 (s, 9H), 1.47 (s, 9H), 3.27-3.31 (m, 6H), 3.41 (q, *J* = 5.8 Hz, 2H), 3.57-3.61 (m, 4H), 4.41 (s, 1H), 4.87 (s, 1H), 6.61 (d, *J* = 2.6 Hz, 1H), 7.02 (dd, *J* = 2.6, 9.0 Hz, 1H), 7.60 (d, *J* = 9.0 Hz, 1H), 7.88 (m, 1H) ppm; **LCMS (ES+)** *m/z* $[M+H]^+$ 506.

***N'*-(3-chloro-2-piperazin-1-yl-6-quinolyl)ethane-1,2-diamine;dihydrochloride (9):**

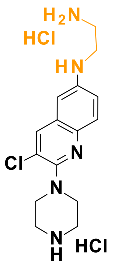

9

The **int-20** (0.07 mmol, 1.0 eq.) was deprotected using **procedure B** with HCl 4M (20 eq.) in 1,4-dioxane (0.6 mL) overnight to give **9** as an orange solid.
Yield: 97%; **Purity (215 nm)**: 99%; **^{1}H NMR (300 MHz, DMSO-*d*$_6$):** δ 3.02-3.08 (m, 2H), 3.22-3.26 (m, 4H), 3.39 (t, *J* = 6.1 Hz, 2H), 3.48-3.52 (m, 4H), 6.84 (d, *J* = 2.1 Hz, 1H), 7.21 (dd, *J* = 2.4, 9.1 Hz, 1H), 7.61 (d, *J* = 9.1 Hz, 1H), 8.18 (s, 1H), 8.32 (br s, 3H), 9.53 (br s, 2H) ppm; **^{13}C NMR (75 MHz, DMSO-*d*$_6$):** δ 37.5, 40.7, 42.6, 46.2, 101.9, 121.9, 121.9, 127.7, 127.8, 135.9, 138.5, 145.5, 152.2 ppm; **LCMS (ES+)** *m/z* $[M+H]^+$ 306; **HRMS (*m/z*)**: $[M+H]^+$ calcd. for $C_{15}H_{21}ClN_5$ 306.1485; found 306.1476.

tert-butyl 4-[6-[[2-(_tert_-butoxycarbonylamino)acetyl]amino]-3-chloro-2-quinolyl]piperazine-1-carboxylate (int-21):

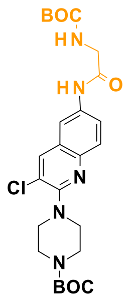

int-21

The **int-21** was obtained using **procedure D**, from *tert*-butyl 4-(3-chloro-6-iodo-2-quinolyl)piperazine-1-carboxylate **int-19** (0.21 mmol, 1.0 eq.), *tert*-butyl *N*-(2-amino-2-oxo-ethyl)carbamate (1.5 eq.), Cs_2CO_3 (1.4 eq.), Xantphos (0.08 eq.), Pd_2dba_3 (0.04 eq.) in dioxane (1.0 mL) at 100 °C overnight. The crude product was purified with a gradient of cyclohexane/EtOAc 100:0 to 50:50 to give **int-21** as a yellow solid.
Yield: 63%; **^{1}H NMR (300 MHz, CD_2Cl_2):** δ 1.47 (s, 9H), 1.48 (s, 9H), 3.36-3.40 (m, 4H), 3.58-3.62 (m, 4H), 3.94 (d, *J* = 6.0 Hz, 2H), 5.36 (br s, 1H), 7.54 (dd, *J* = 2.4, 9.0 Hz, 1H), 7.74 (d, *J* = 9.0 Hz, 1H), 8.00 (s, 1H), 8.06 (d, *J* = 1.8 Hz, 1H), 8.40 (br s, 1H) ppm; **LCMS (ES+)** *m/z* $[M+H]^+$ 520.

2-amino-_N_-(3-chloro-2-piperazin-1-yl-6-quinolyl)acetamide;dihydrochloride (10):

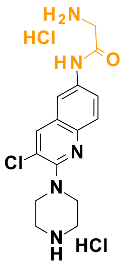

10

The **int-20** (0.13 mmol, 1.0 eq.) was deprotected using **procedure B** with HCl 4M (20 eq.) in 1,4-dioxane (1.3 mL) overnight to give **10** as a yellow solid.
Yield: 100%; **Purity (215 nm)**: 99%; **^{1}H NMR (300 MHz, DMSO-d_6):** δ 3.24-3.29 (m, 4H), 3.58-3.62 (m, 4H), 3.84-3.91 (m, 2H), 7.79 (d, *J* = 9.0 Hz, 1H), 7.86 (dd, *J* = 1.9, 9.0 Hz, 1H), 8.25 (d, *J* = 1.8 Hz, 1H), 8.36 (br s, 3H), 8.48 (s, 1H), 9.56 (br s, 2H), 11.22 (br s, 1H) ppm; **^{13}C NMR (75 MHz, DMSO-d_6):** δ 41.1, 42.5, 45.9, 114.4, 122.2, 123.4, 126.0, 127.9, 135.6, 137.7, 141.5, 154.7, 165.1 ppm; **LCMS (ES+)** *m/z* $[M+H]^+$ 320; **HRMS (_m/z_)**: $[M+H]^+$ calcd. for $C_{15}H_{19}ClN_5O$ 320.1278; found 320.1270.

tert-butyl 4-[6-[3-(_tert_-butoxycarbonylamino)propylamino]-3-chloro-2-quinolyl]piperazine-1-carboxylate (int-22):

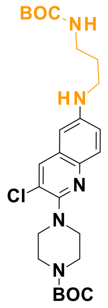

int-22

The **int-22** was obtained using **procedure D**, from *tert*-butyl 4-(3-chloro-6-iodo-2-quinolyl)piperazine-1-carboxylate **int-19** (0.21 mmol, 1.0 eq.), *tert-butyl* N-(3-aminopropyl)carbamate (1.5 eq.), Cs_2CO_3 (1.4 eq.), Xantphos (0.08 eq.), Pd_2dba_3 (0.05 eq.) in dioxane (1.0 mL) at 100 °C overnight. The crude product was purified with a gradient of cyclohexane/EtOAc 100:0 to 60:40 to give **int-22** as a yellow oil.
Yield: 43%; **^{1}H NMR (300 MHz, CD_2Cl_2):** δ 1.43 (s, 9H), 1.47 (s, 9H), 1.81 (q, *J* = 6.6 Hz, 2H), 3.20-3.31 (m, 8H), 3.57-3.61 (m, 4H), 4.29 (s, 1H), 4.70 (s, 1H), 6.61 (d, *J* = 2.6 Hz, 1H), 7.03 (dd, *J* = 2.6, 9.0 Hz, 1H), 7.60 (d, *J* = 9.0 Hz, 1H), 7.87 (s, 1H) ppm; **LCMS (ES+)** *m/z* $[M+H]^+$ 520.

N'-(3-chloro-2-piperazin-1-yl-6-quinolyl)propane-1,3-diamine;dihydrochloride (11):

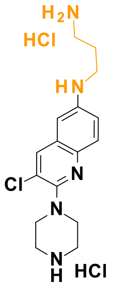

11

The **int-22** (0.07 mmol, 1.0 eq.) was deprotected using **procedure B** with HCl 4M (27 eq.) in 1,4-dioxane (0.8 mL) overnight to give **11** as an orange solid.

Yield: 77%; **Purity (215 nm)**: 99%; **^{1}H NMR (300 MHz, DMSO-*d*$_6$):** δ 1.94-2.01 (m, 2H), 2.88-2.95 (m, 2H), 3.23-3.30 (m, 6H), 3.53-3.58 (m, 4H), 7.15-7.19 (m, 1H), 7.43 (d, *J* = 8.5 Hz, 1H), 7.71 (d, *J* = 9.0 Hz, 1H), 8.18 (br s, 3H), 8.31 (s, 1H), 9.59 (br s, 2H) ppm; **^{13}C NMR (75 MHz, DMSO-*d*$_6$):** δ 25.3, 36.6, 42.5, 46.1, 122.1, 122.9, 127.1, 128.0, 136.6, 139.8, 141.9, 153.2 ppm; **LCMS (ES+)** *m/z* [M+H]$^+$ 320; **HRMS (*m/z*)**: [M+H]$^+$ calcd. for $C_{16}H_{23}ClN_5$ 320.1642; found 320.1638.

- Synthesis of compounds **12-13**:

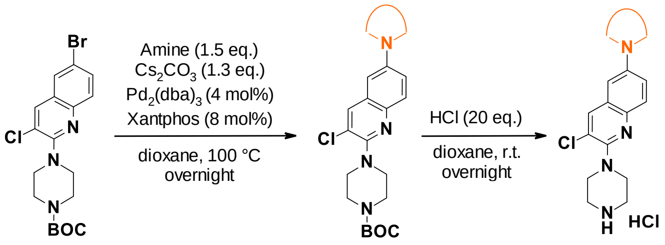

int-9 int-23-24 12-13

***tert*-butyl 4-[6-[(3*R*)-3-(*tert*-butoxycarbonylamino)pyrrolidin-1-yl]-3-chloro-2-quinolyl]piperazine-1-carboxylate (int-23):**

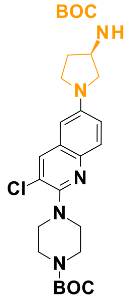

int-23

The **int-23** was obtained using **procedure D**, from *tert*-butyl 4-(3-chloro-6-bromo-2-quinolyl)piperazine-1-carboxylate **int-9** (0.23 mmol, 1.0 eq.), *tert*-butyl *N*-[(3*R*)-pyrrolidin-3-yl]carbamate (1.5 eq.), Cs_2CO_3 (1.3 eq.), Xantphos (0.08 eq.), Pd_2dba_3 (0.04 eq.) in dioxane (1.4 mL) at 100 °C overnight. The crude product was purified with a gradient of cyclohexane/EtOAc 100:0 to 80:20 to give **int-23** as a yellow oil.

Yield: 46%; **^{1}H NMR (300 MHz, CD_2Cl_2):** δ 1.43 (s, 9H), 1.47 (s, 9H), 1.92-2.02 (m, 1H), 2.26-2.37 (m, 1H), 3.21-3.68 (m, 12H), 4.34 (s, 1H), 4.81 (s, 1H), 6.55 (d, *J* = 2.7 Hz, 1H), 7.10 (dd, *J* = 2.7, 9.2 Hz, 1H), 7.68 (d, *J* = 9.2 Hz, 1H), 7.90 (s, 1H) ppm; **LCMS (ES+)** *m/z* [M+H]$^+$ 532.

(3*R*)-1-(3-chloro-2-piperazin-1-yl-6-quinolyl)pyrrolidin-3-amine;dihydrochloride (12):

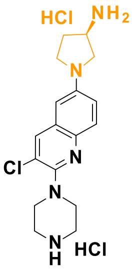

12

The **int-23** (0.09 mmol, 1.0 eq.) was deprotected using **procedure B** with HCl 4M (20 eq.) in 1,4-dioxane (0.6 mL) overnight to give **12** as an orange solid.

Yield: 100%; **Purity (215 nm)**: 99%; **^{1}H NMR (300 MHz, DMSO-*d*$_6$):** δ 2.13-2.22 (m, 1H), 2.29-2.41 (m, 1H), 3.22-3.27 (m, 4H), 3.35-3.66 (m, 8H), 3.93-4.00 (m, 1H), 6.77 (d, *J* = 2.5 Hz, 1H), 7.20 (dd, *J* = 2.6, 9.2 Hz, 1H), 7.71 (d, *J* = 9.1 Hz, 1H), 8.28 (s, 1H), 8.56 (br s, 3H), 9.55 (br s, 2H) ppm; **^{13}C NMR (75 MHz, DMSO-*d*$_6$):** δ 29.2, 42.6, 45.6, 46.2, 49.5, 51.4, 103.7, 119.2, 122.1, 127.5, 127.9, 136.0, 137.7, 144.7, 152.2 ppm; **LCMS (ES+)** *m/z* [M+H]$^+$ 332; **HRMS (*m/z*)**: [M+H]$^+$ calcd. for $C_{17}H_{23}ClN_5$ 332.1642; found 332.1636.

tert-butyl 4-[6-[(3S)-3-(tert-butoxycarbonylamino)pyrrolidin-1-yl]-3-chloro-2-quinolyl]piperazine-1-carboxylate (int-24):

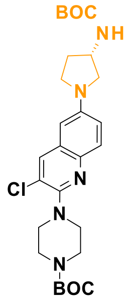

int-24

The **int-24** was obtained using **procedure C**, from *tert*-butyl 4-(3-chloro-6-bromo-2-quinolyl)piperazine-1-carboxylate **int-9** (0.35 mmol, 1.0 eq.), *tert*-butyl *N*-[(3*S*)-pyrrolidin-3-yl]carbamate (1.5 eq.), Cs_2CO_3 (1.3 eq.), Xantphos (0.08 eq.), Pd_2dba_3 (0.04 eq.) in dioxane (2.0 mL) at 100 °C overnight. The crude product was purified with a gradient of cyclohexane/EtOAc 100:0 to 60:40 to give **int-24** as a yellow solid.
Yield: 81%; **^{1}H NMR (300 MHz, CD_2Cl_2):** δ 1.43 (s, 9H), 1.47 (s, 9H), 1.92-2.03 (m, 1H), 2.26-2.38 (m, 1H), 3.21-3.31 (m, 5H), 3.37-3.67 (m, 7H), 4.36 (s, 1H), 4.81 (s, 1H), 6.55 (d, *J* = 2.6 Hz, 1H), 7.09 (dd, *J* = 2.7, 9.2 Hz, 1H), 7.68 (d, *J* = 9.1 Hz, 1H), 7.90 (s, 1H) ppm; **LCMS (ES+)** *m/z* $[M+H]^+$ 532.

(3S)-1-(3-chloro-2-piperazin-1-yl-6-quinolyl)pyrrolidin-3-amine;dihydrochloride (13):

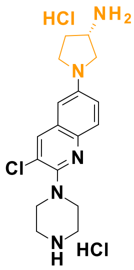

13

The **int-24** (0.29 mmol, 1.0 eq.) was deprotected using **procedure B** with HCl 4M (20 eq.) in 1,4-dioxane (1.8 mL) overnight to give **13** as a red solid.
Yield: 95%; **Purity (215 nm)**: 99%; **^{1}H NMR (300 MHz, DMSO-d_6):** δ 2.11-2.22 (m, 1H), 2.29-2.42 (m, 1H), 3.22-3.27 (m, 4H), 3.35-3.66 (m, 8H), 3.92-4.00 (m, 1H), 6.77 (d, *J* = 2.4 Hz, 1H), 7.21 (dd, *J* = 2.5, 9.2 Hz, 1H), 7.72 (d, *J* = 9.2 Hz, 1H), 8.28 (s, 1H), 8.57 (br s, 3H), 9.57 (br s, 2H) ppm; **^{13}C NMR (75 MHz, DMSO-d_6):** δ 29.2, 42.6, 45.6, 46.2, 49.5, 51.4, 103.8, 119.3, 122.1, 127.5, 127.9, 136.0, 137.7, 144.7, 152.2 ppm; **LCMS (ES+)** *m/z* $[M+H]^+$ 332; **HRMS (*m/z*)**: $[M+H]^+$ calcd. for $C_{17}H_{23}ClN_5$ 332.1642; found 332.1646.

NMR spectra of PyrPip compounds 1’-13

Compound **1'** NMR ^{1}H DMSO-d_6

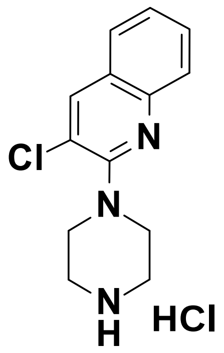

1'

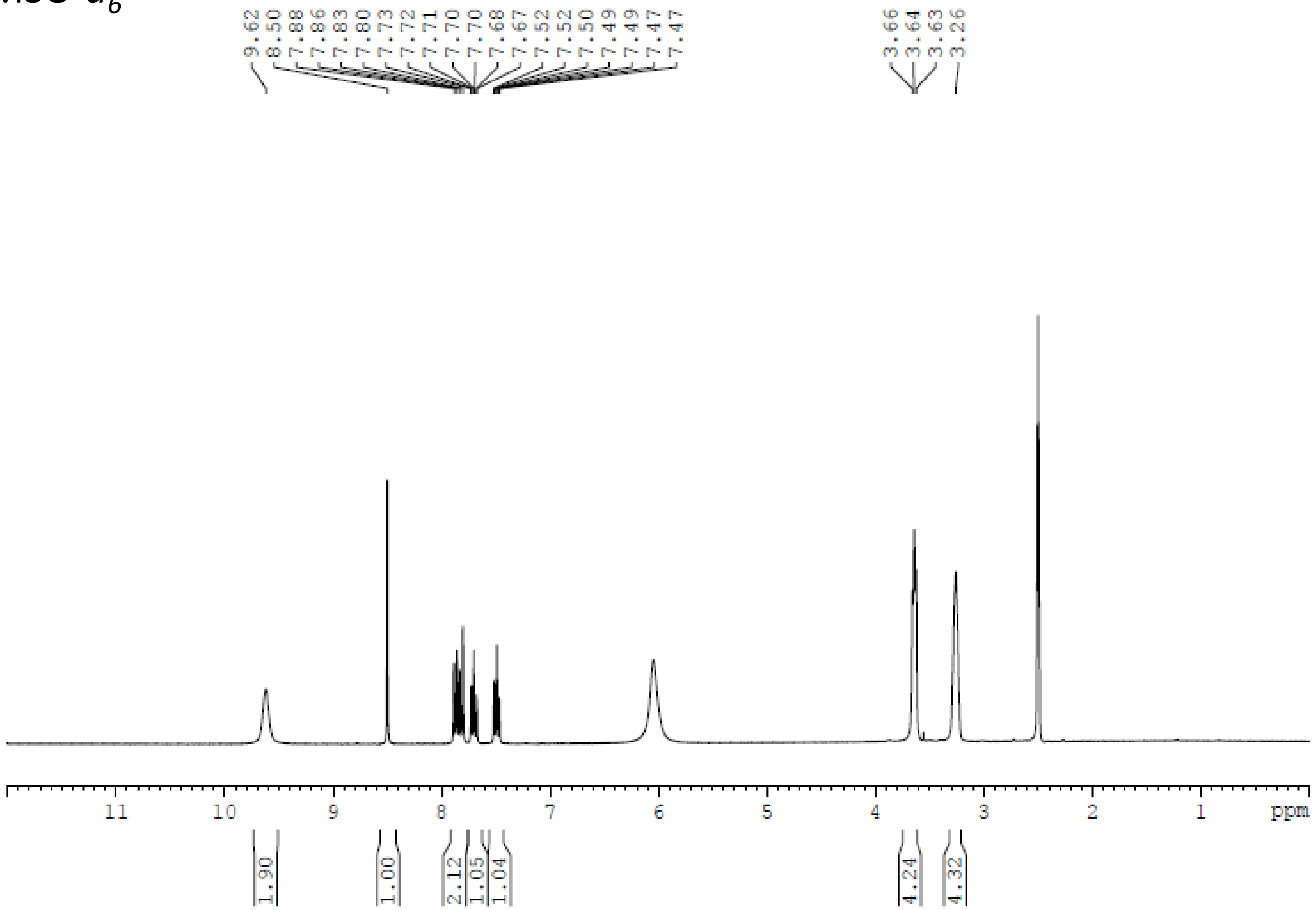

Compound **1'** NMR ^{13}C DMSO-d_6

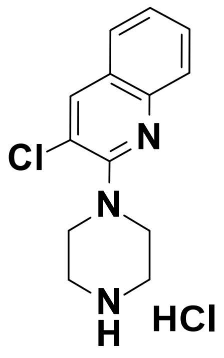

1'

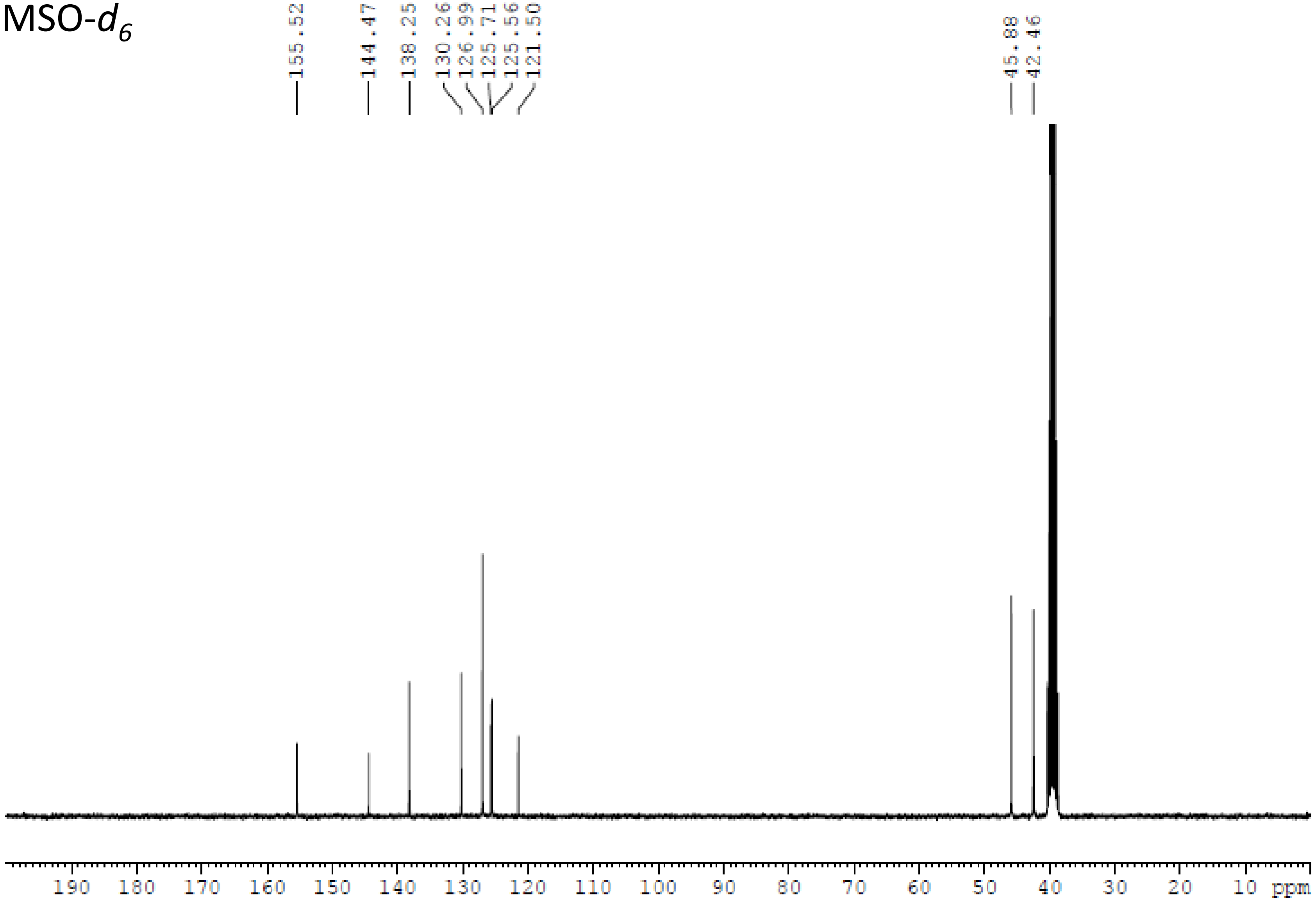

Compound **2** NMR ^{1}H DMSO-d_6

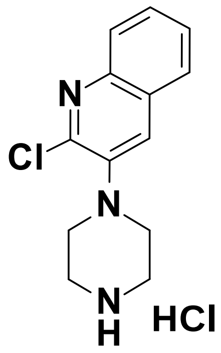

2

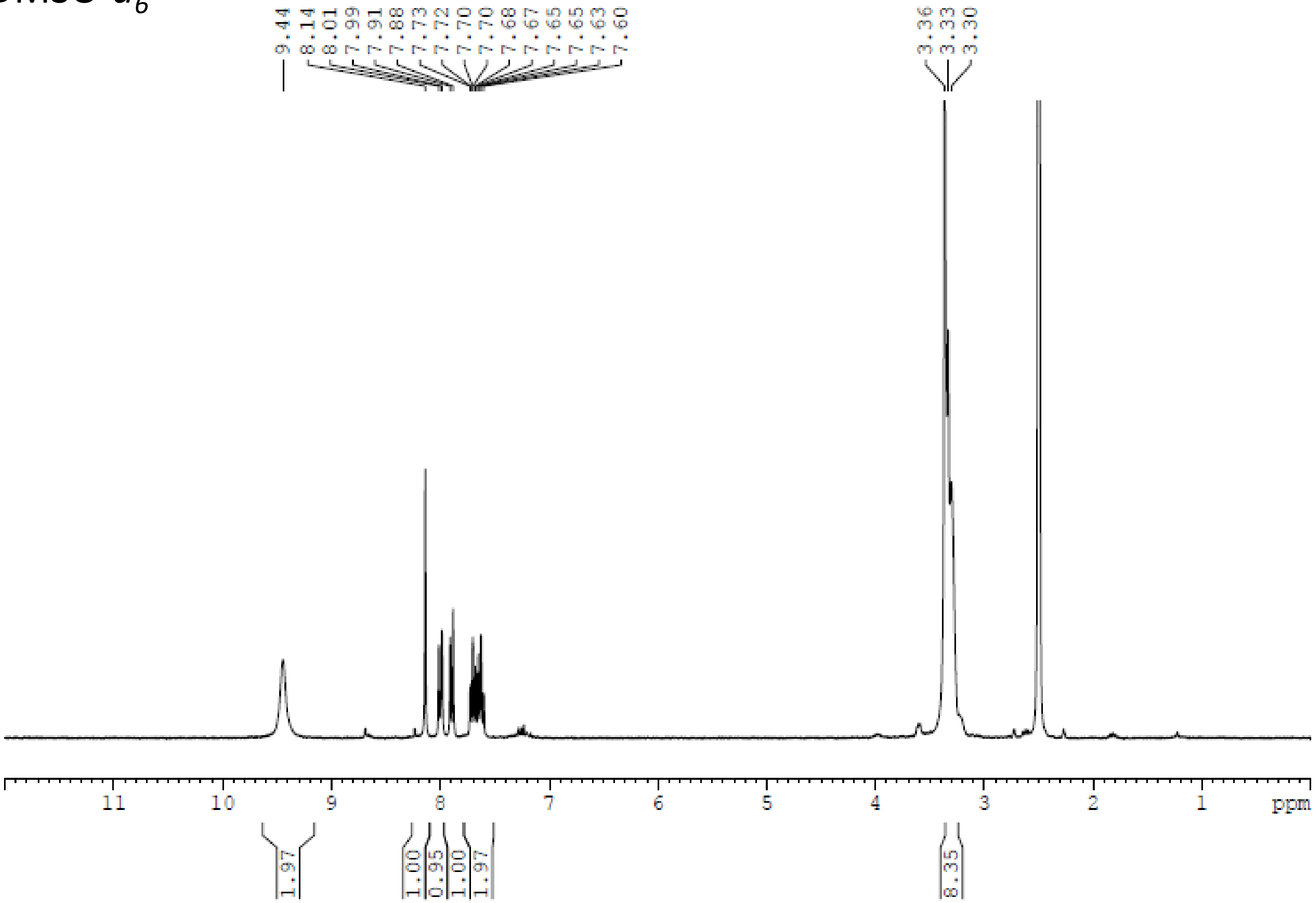

Compound **2** NMR ^{13}C DMSO-d_6

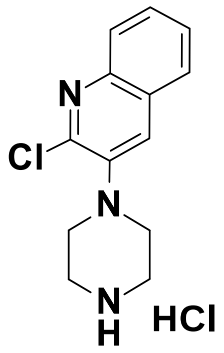

2

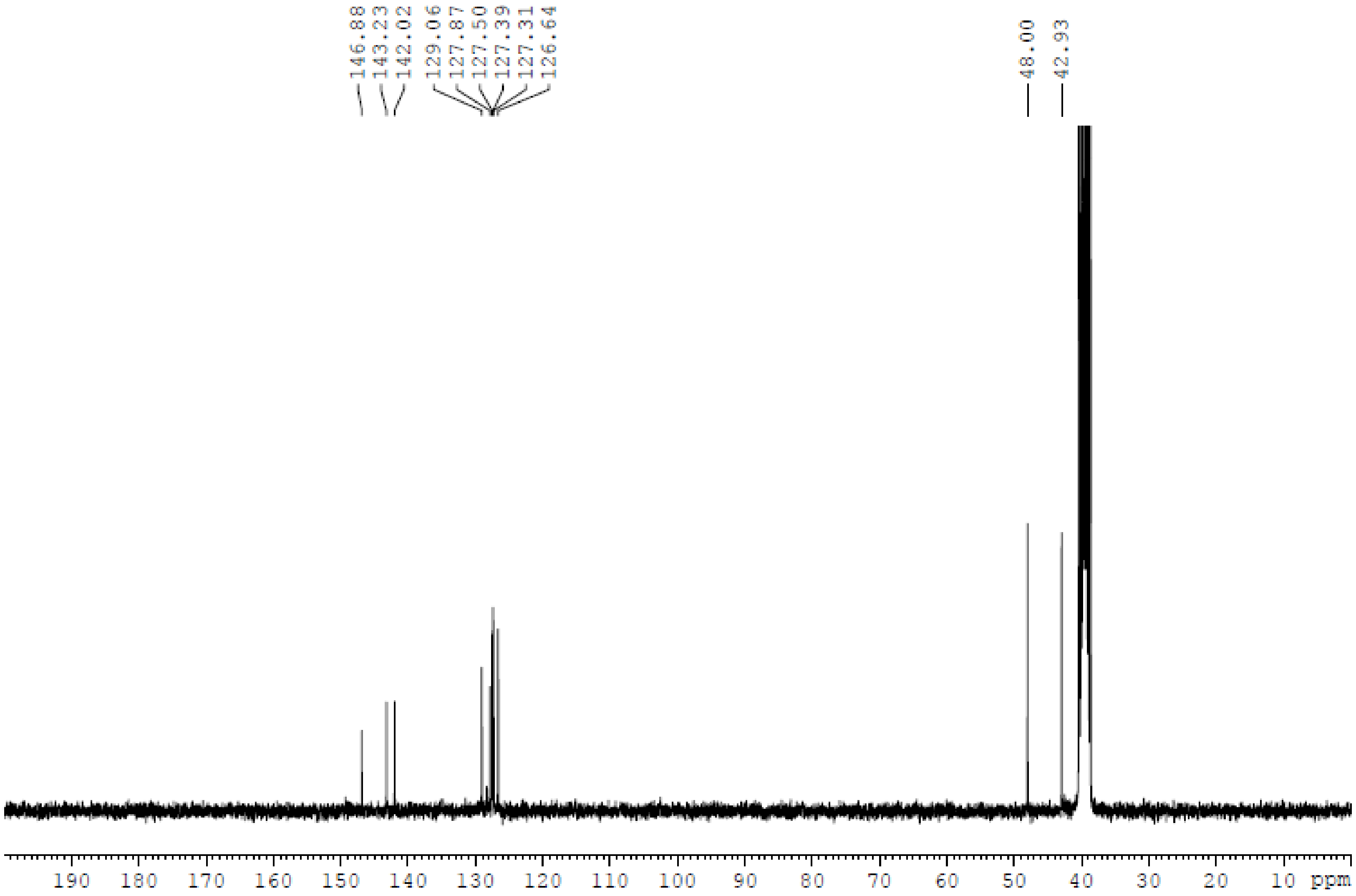

Compound **3** NMR ^{1}H DMSO-d_6

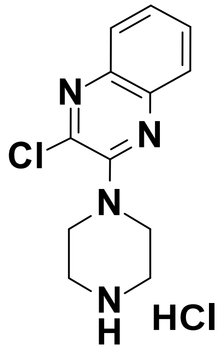

3

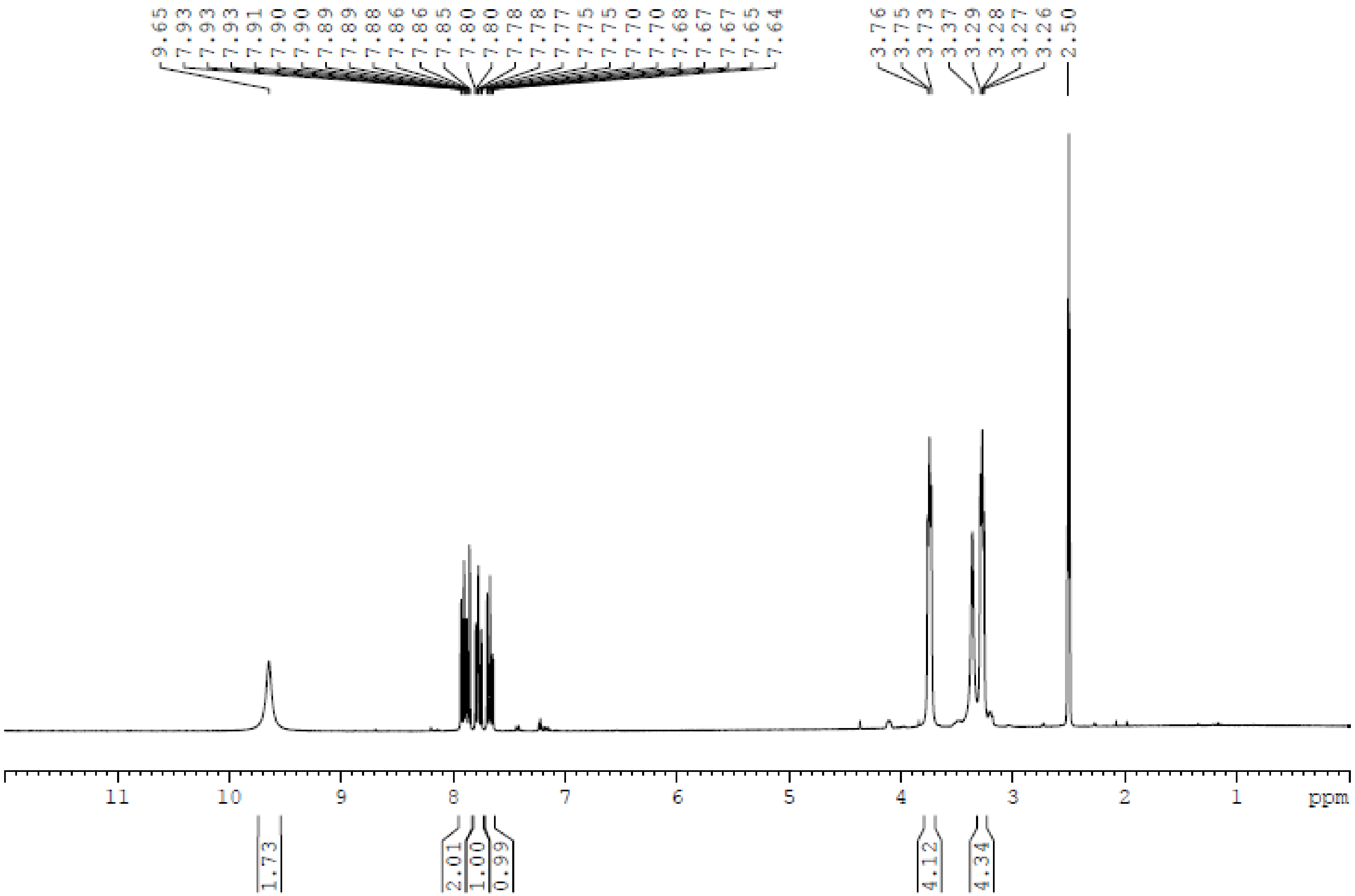

Compound **3** NMR ^{13}C DMSO-d_6

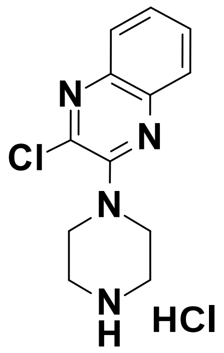

3

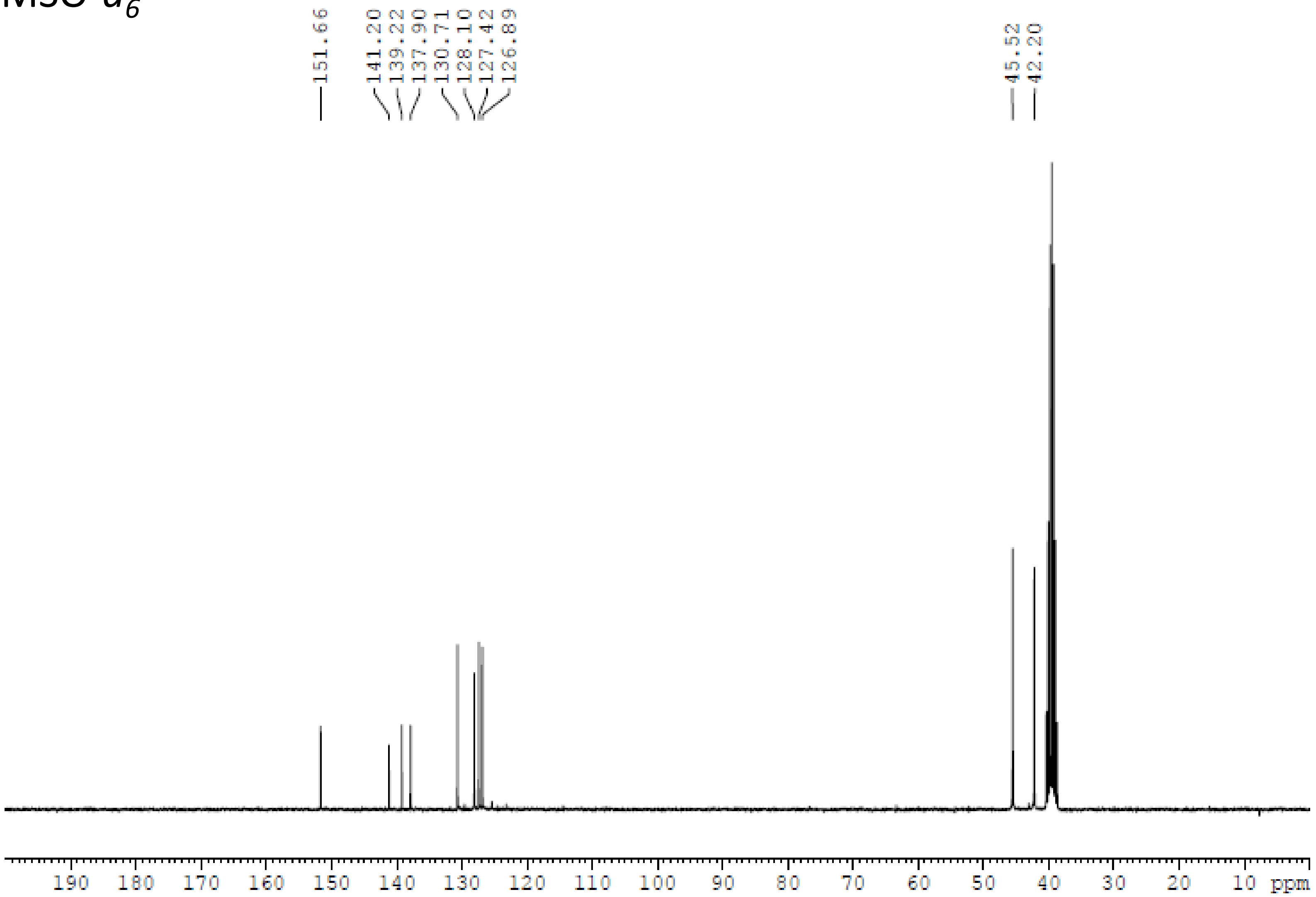

Compound **4** NMR ^{1}H DMSO-d_6

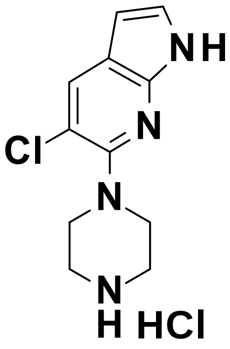

4

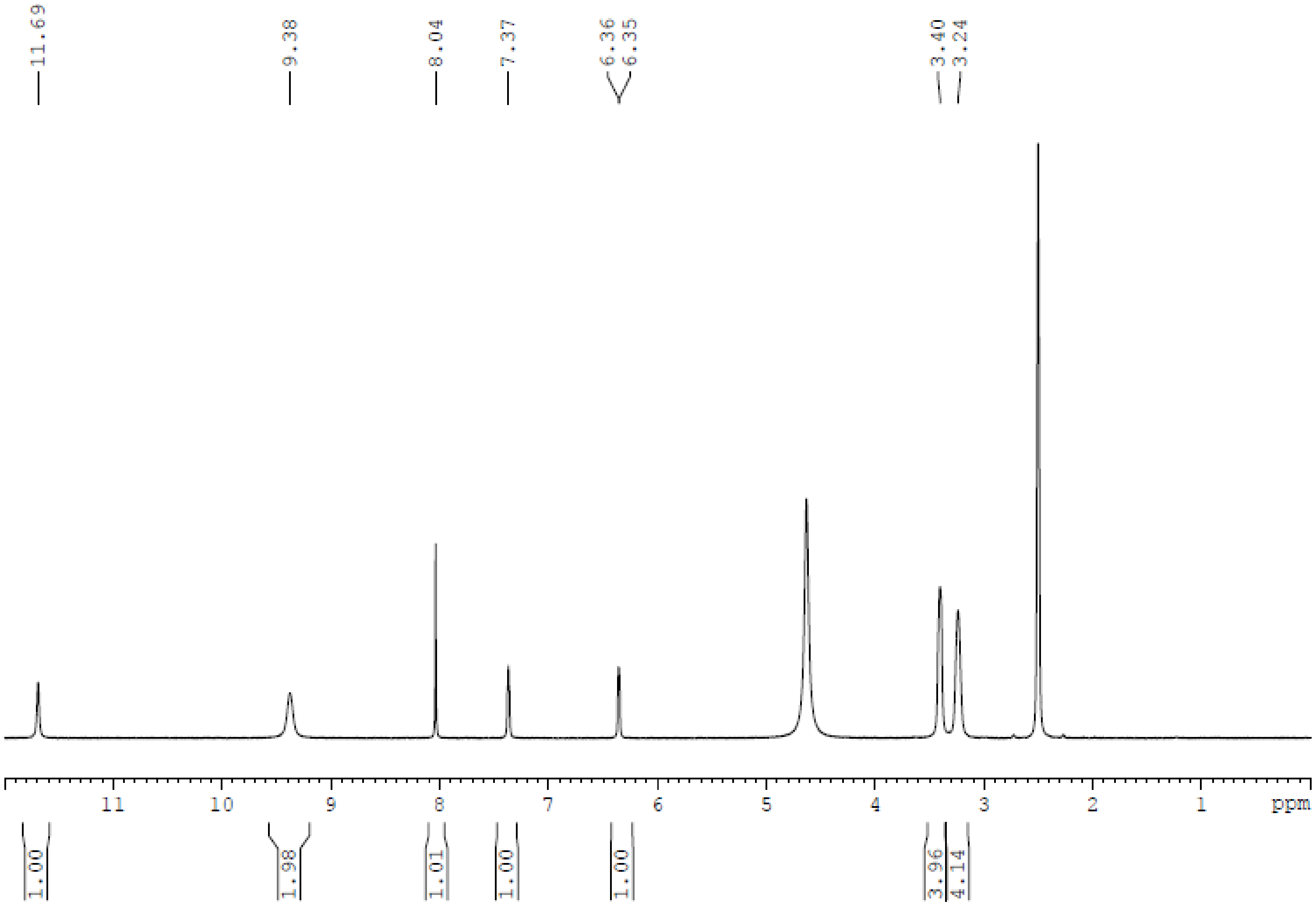

Compound **4** NMR ^{13}C DMSO-d_6

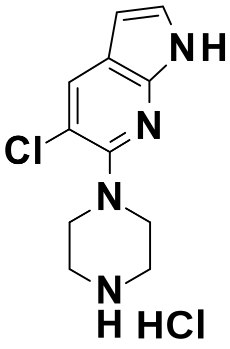

4

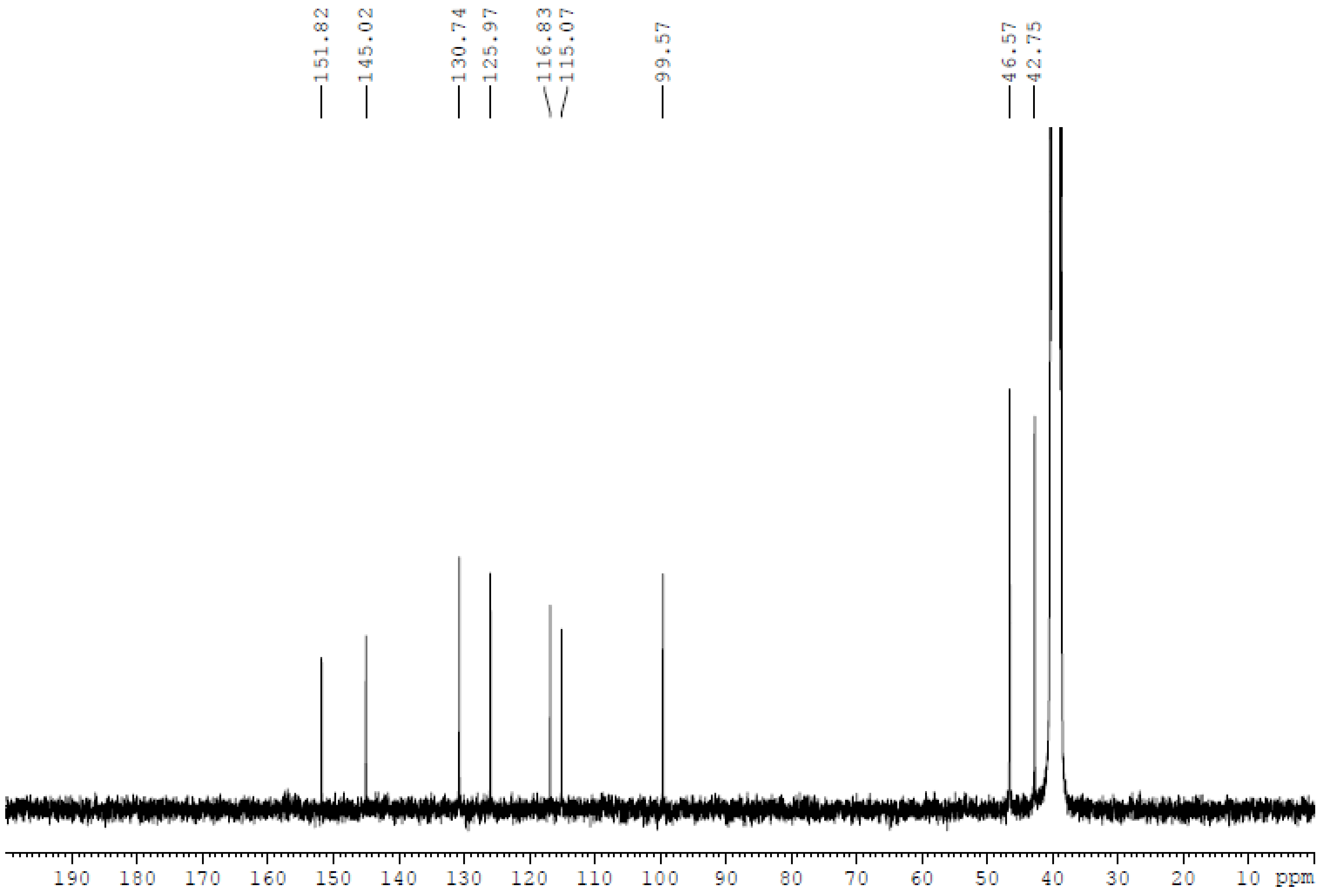

Compound **5** NMR ^1H DMSO-d_6

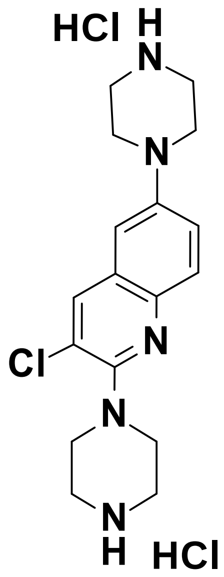

5

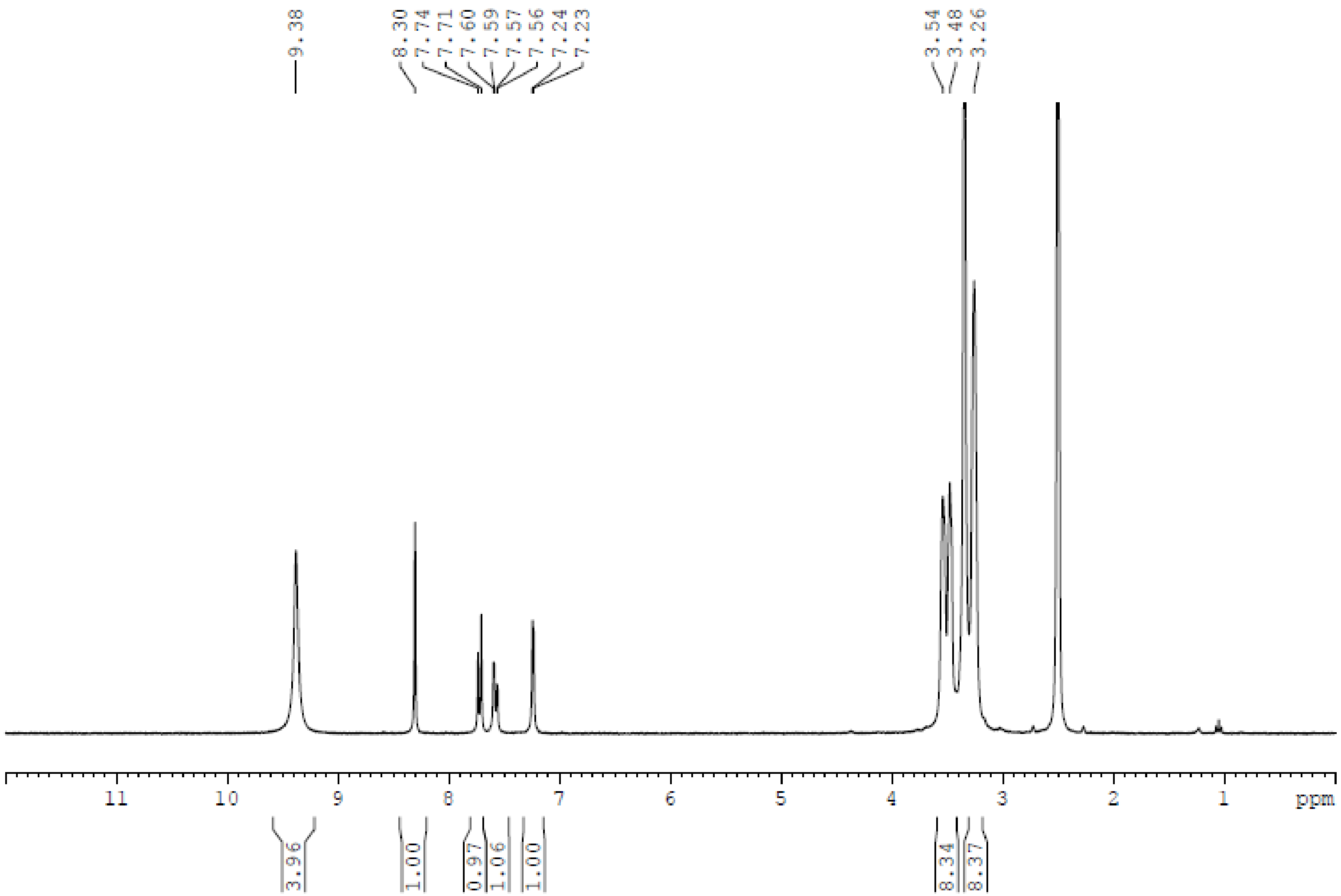

Compound **5** NMR ^{13}C DMSO-d_6

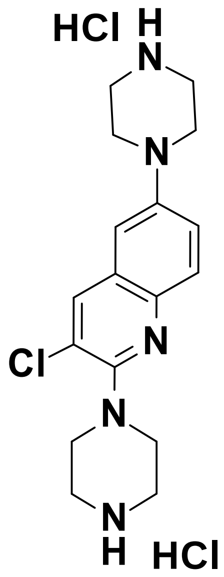

5

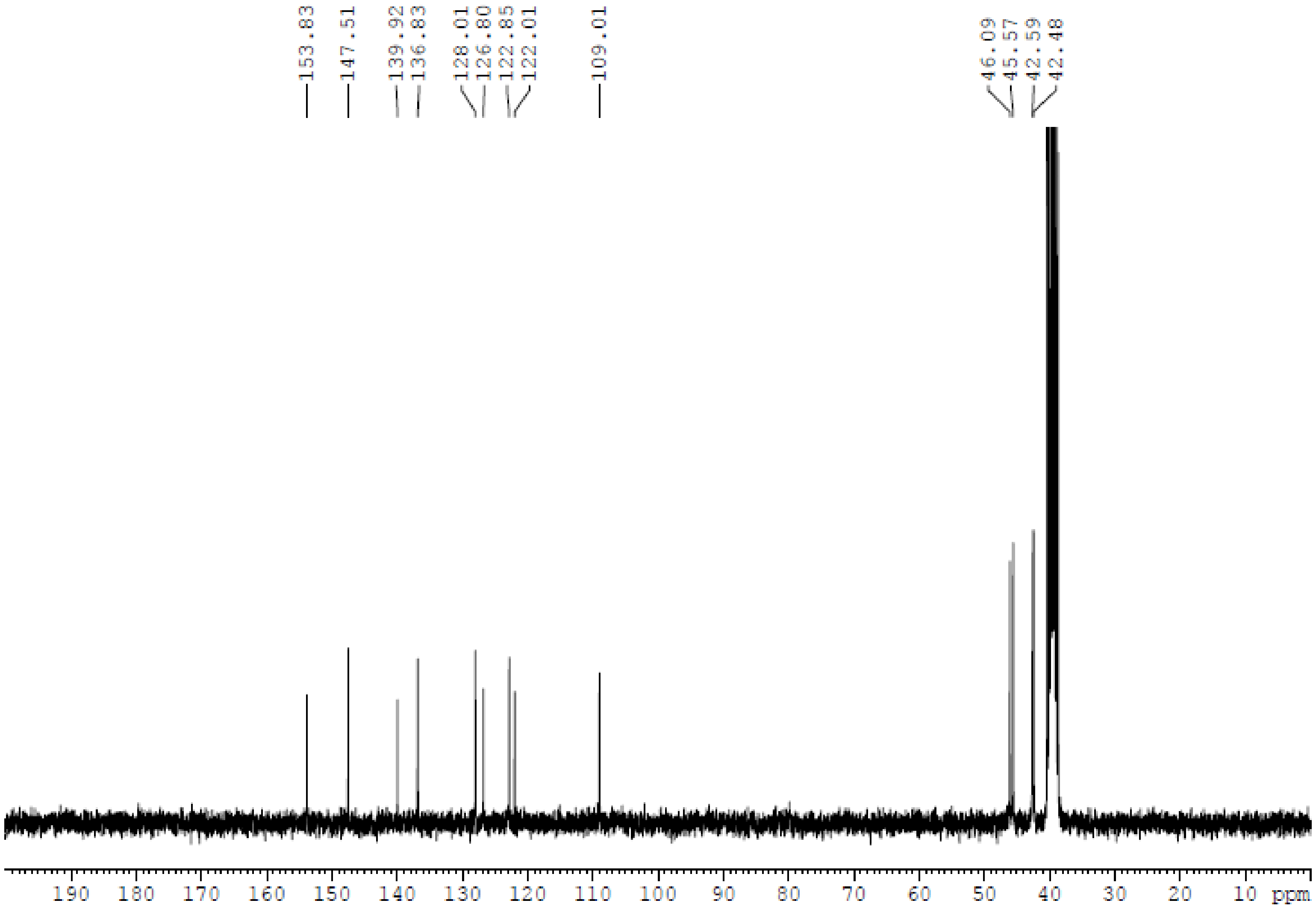

Compound **6** NMR ^{1}H DMSO-d_6

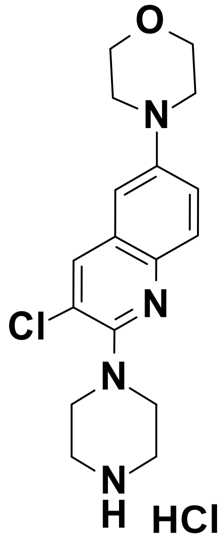

6

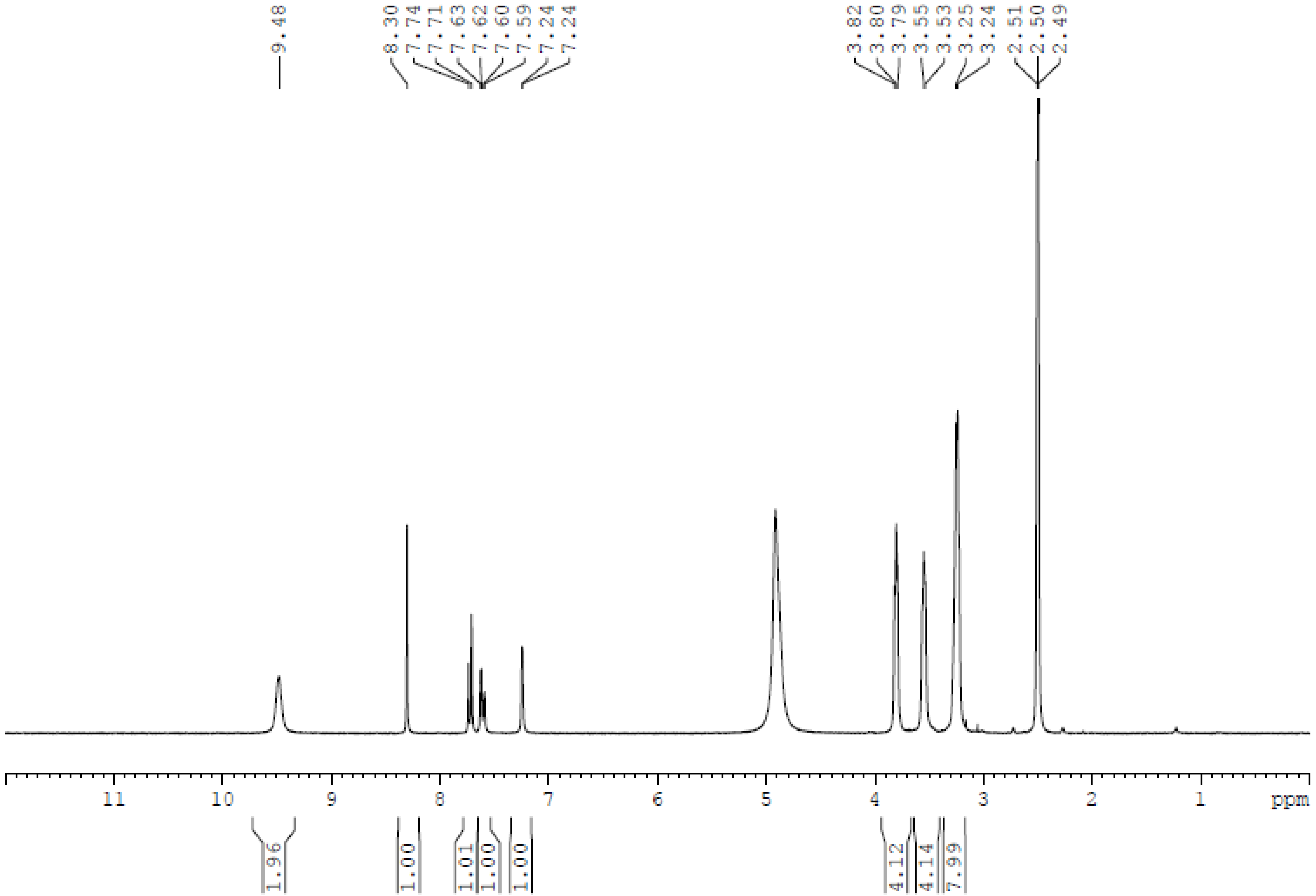

Compound **6** NMR ^{13}C DMSO-d_6

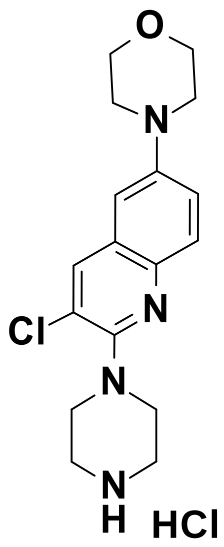

6

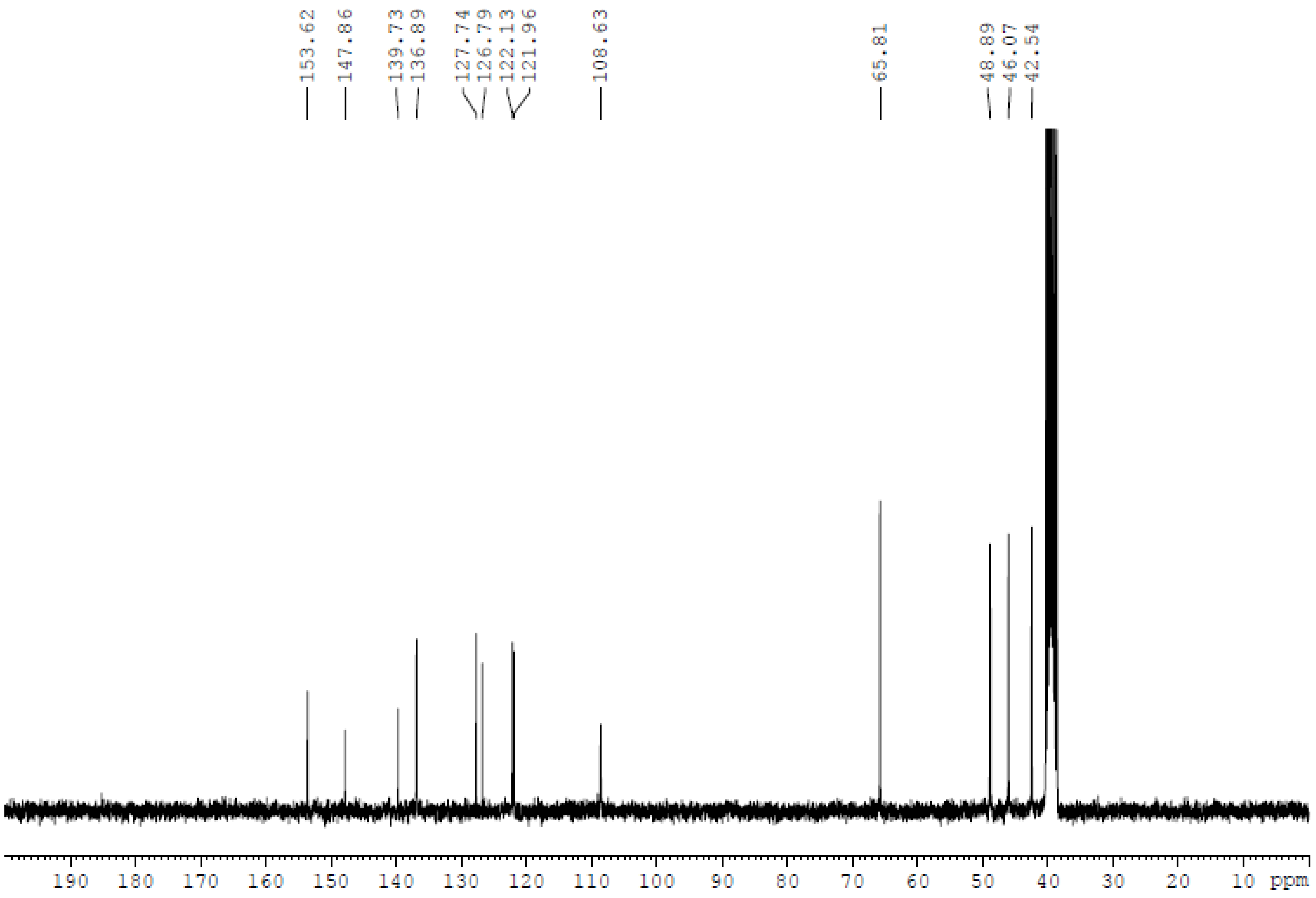

Compound **7** NMR ^{1}H DMSO-d_6

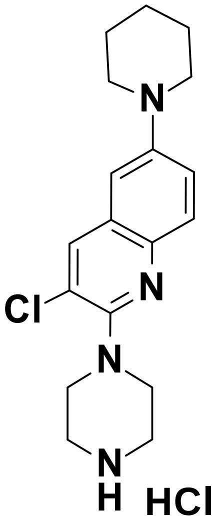

7

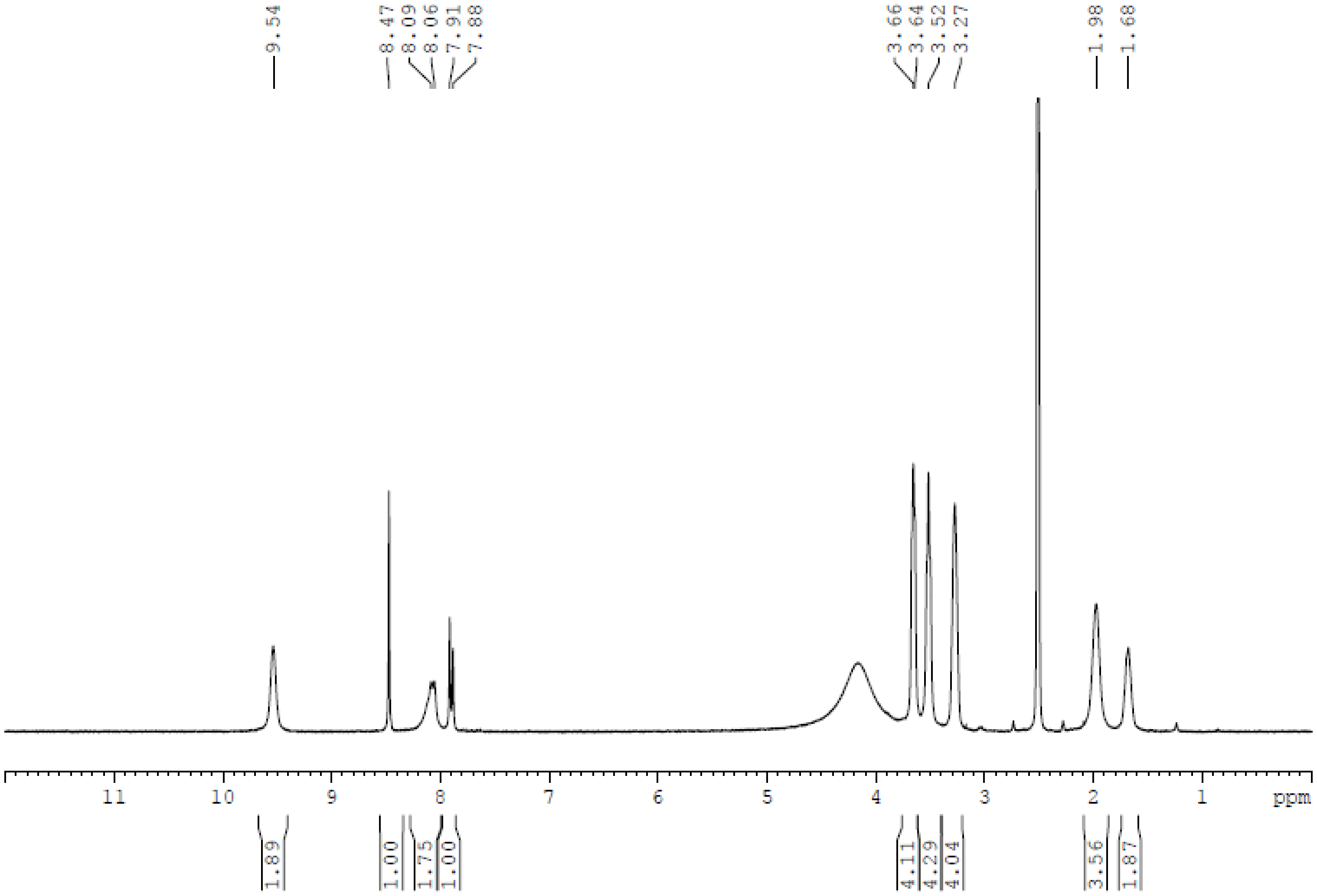

Compound **7** NMR ^{13}C DMSO-d_6

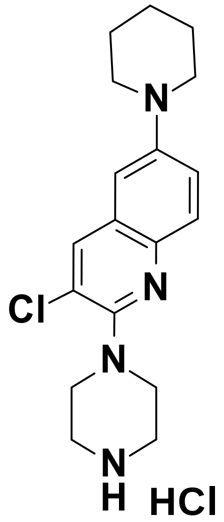

7

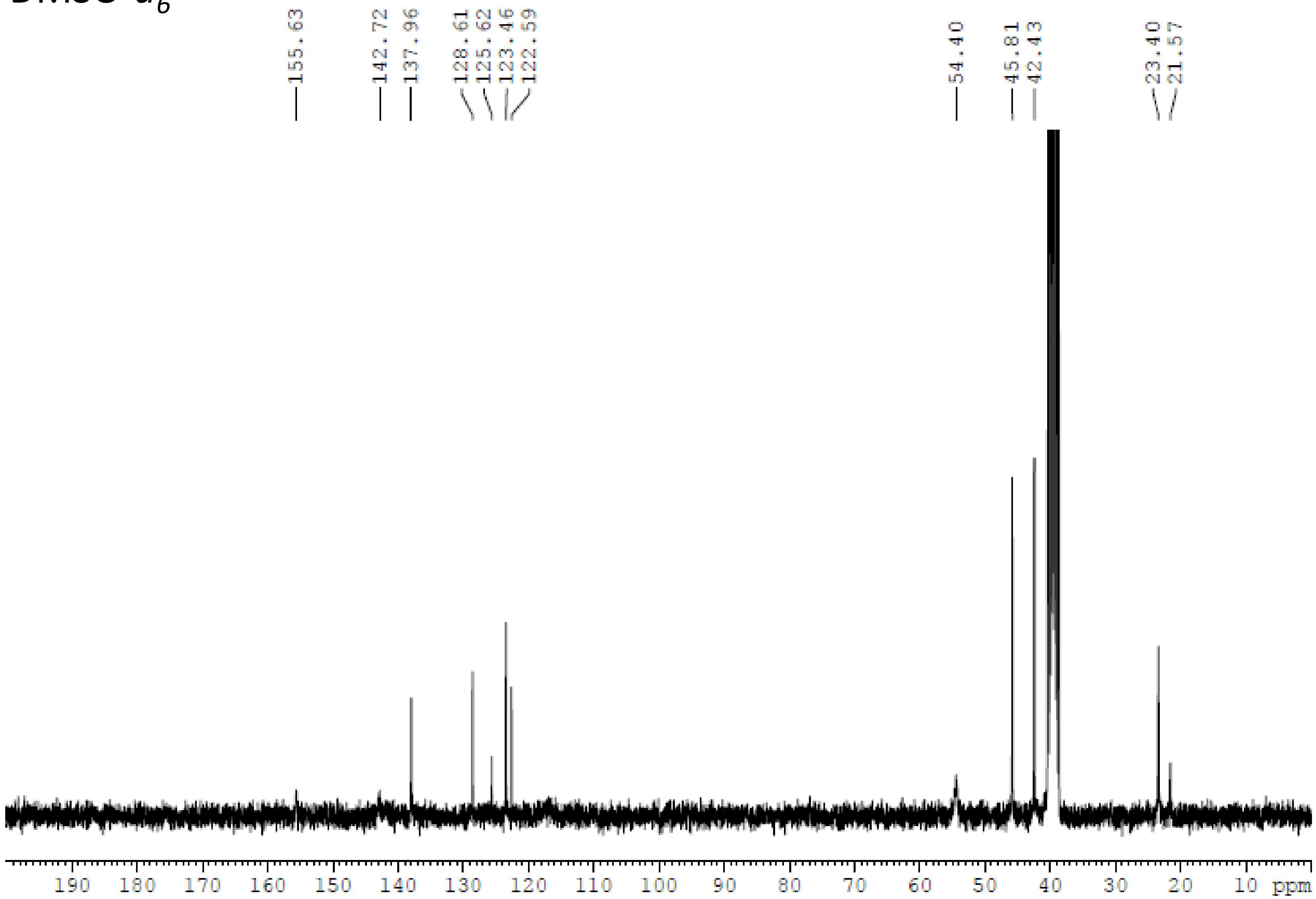

Compound **8** NMR ^{1}H DMSO-d_6

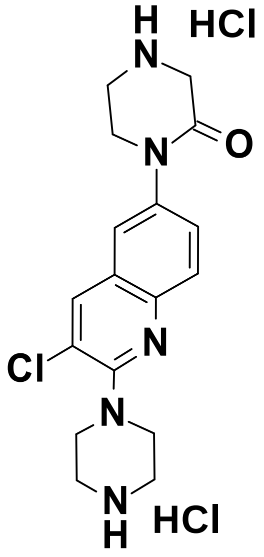

8

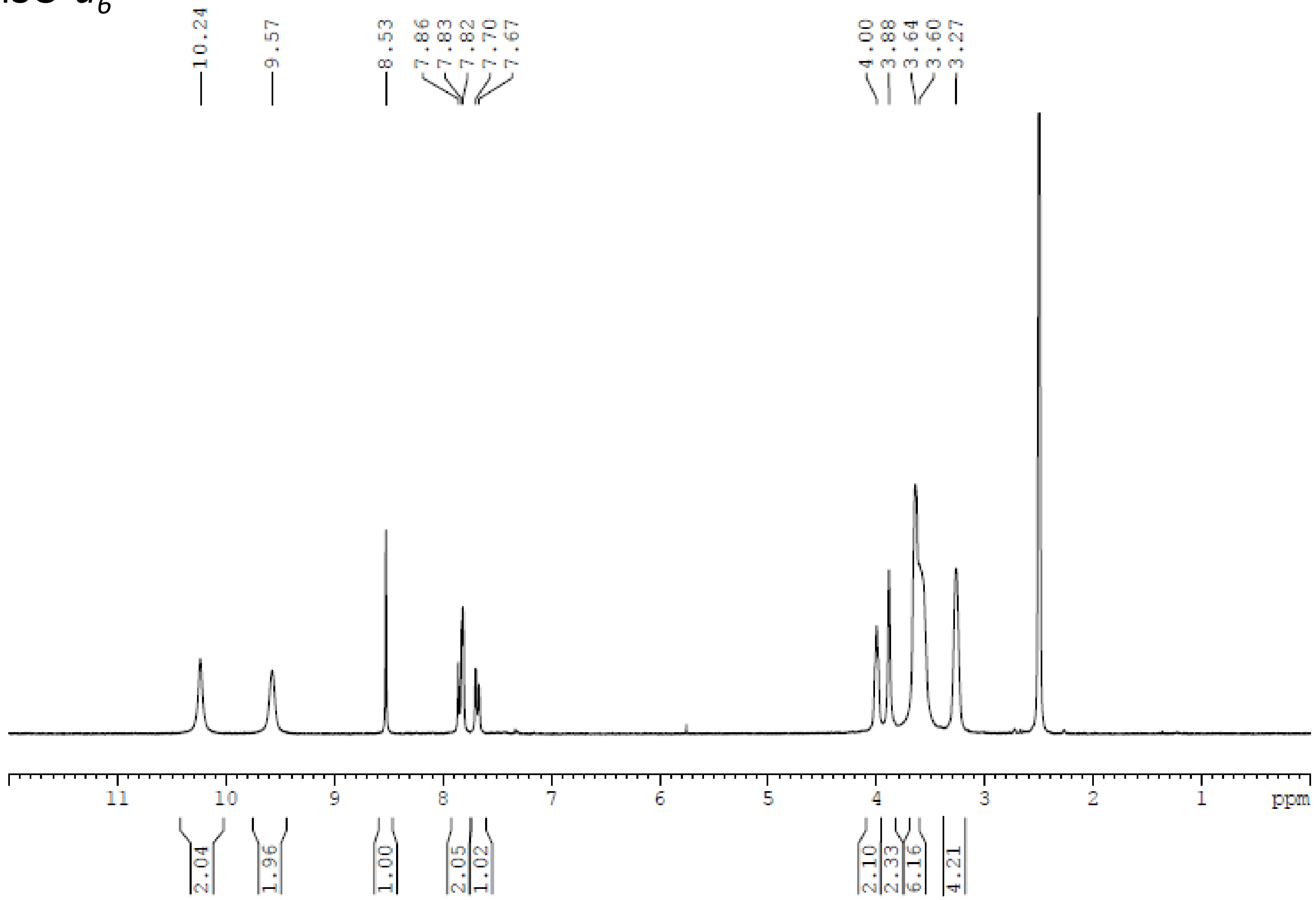

Compound **8** NMR ^{13}C DMSO-d_6

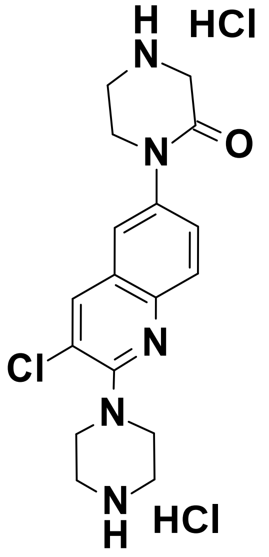

8

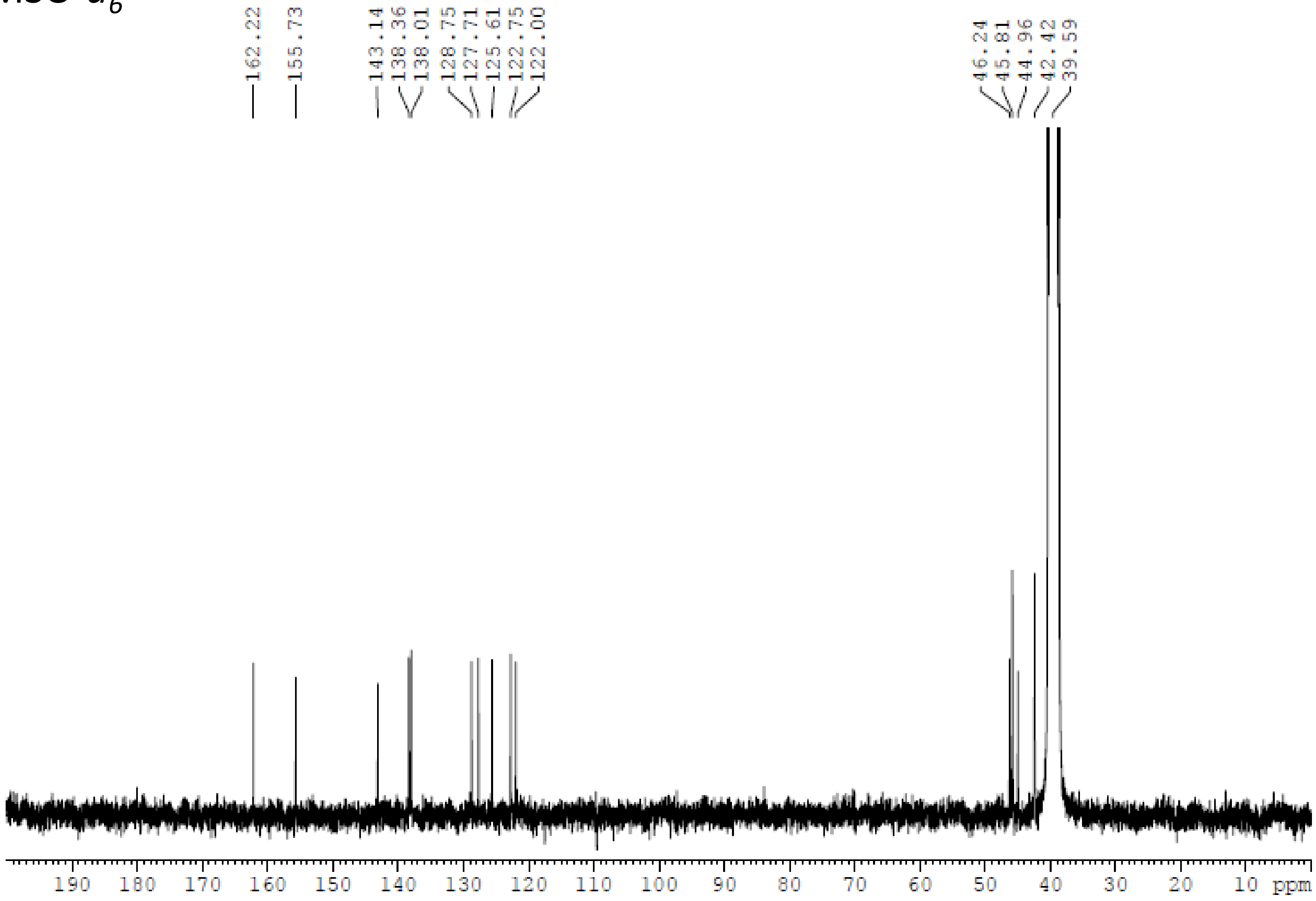

Compound **9** NMR ^1H DMSO-d_6

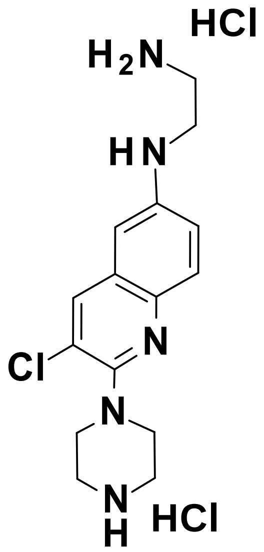

9

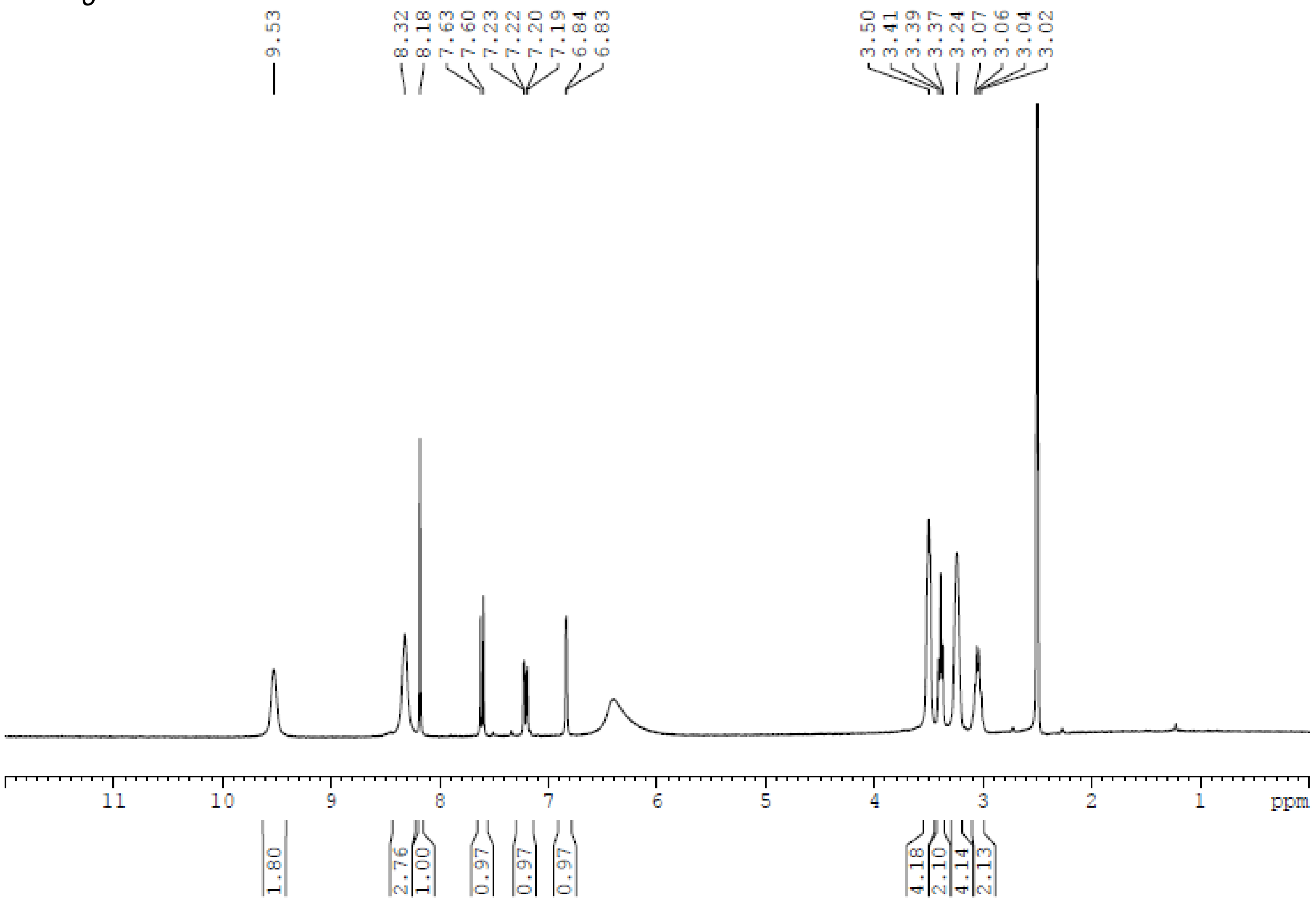

Compound **9** NMR ^{13}C DMSO-d_6

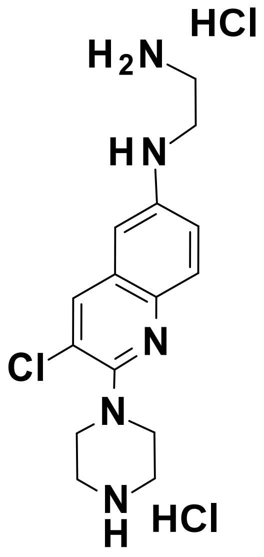

9

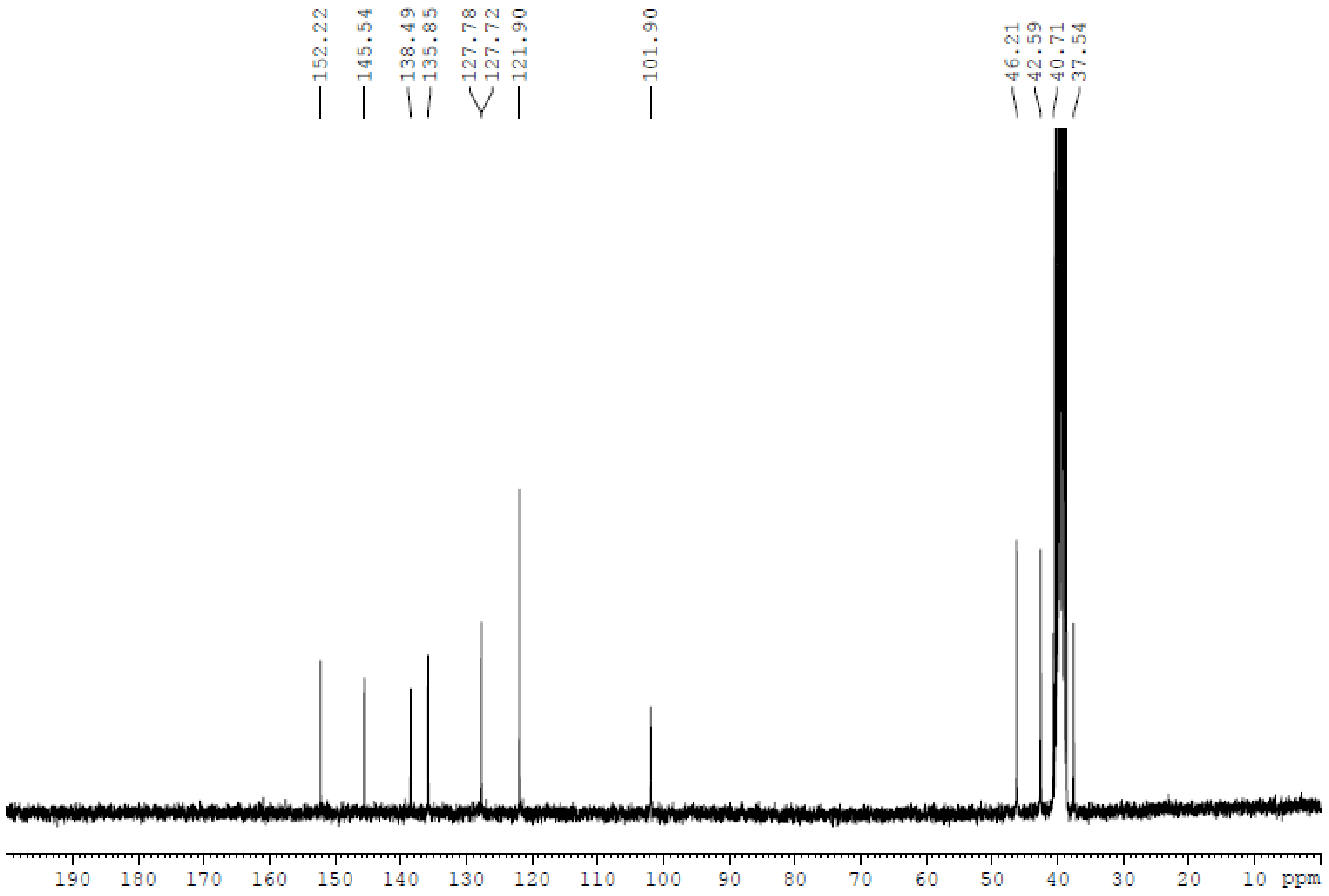

Compound **10** NMR ^{1}H DMSO-d_6

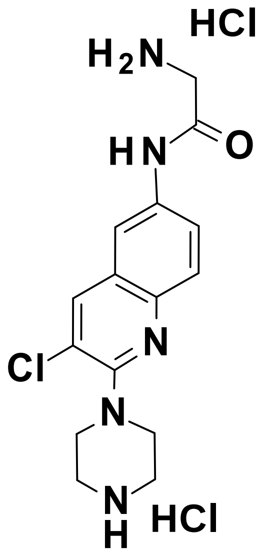

10

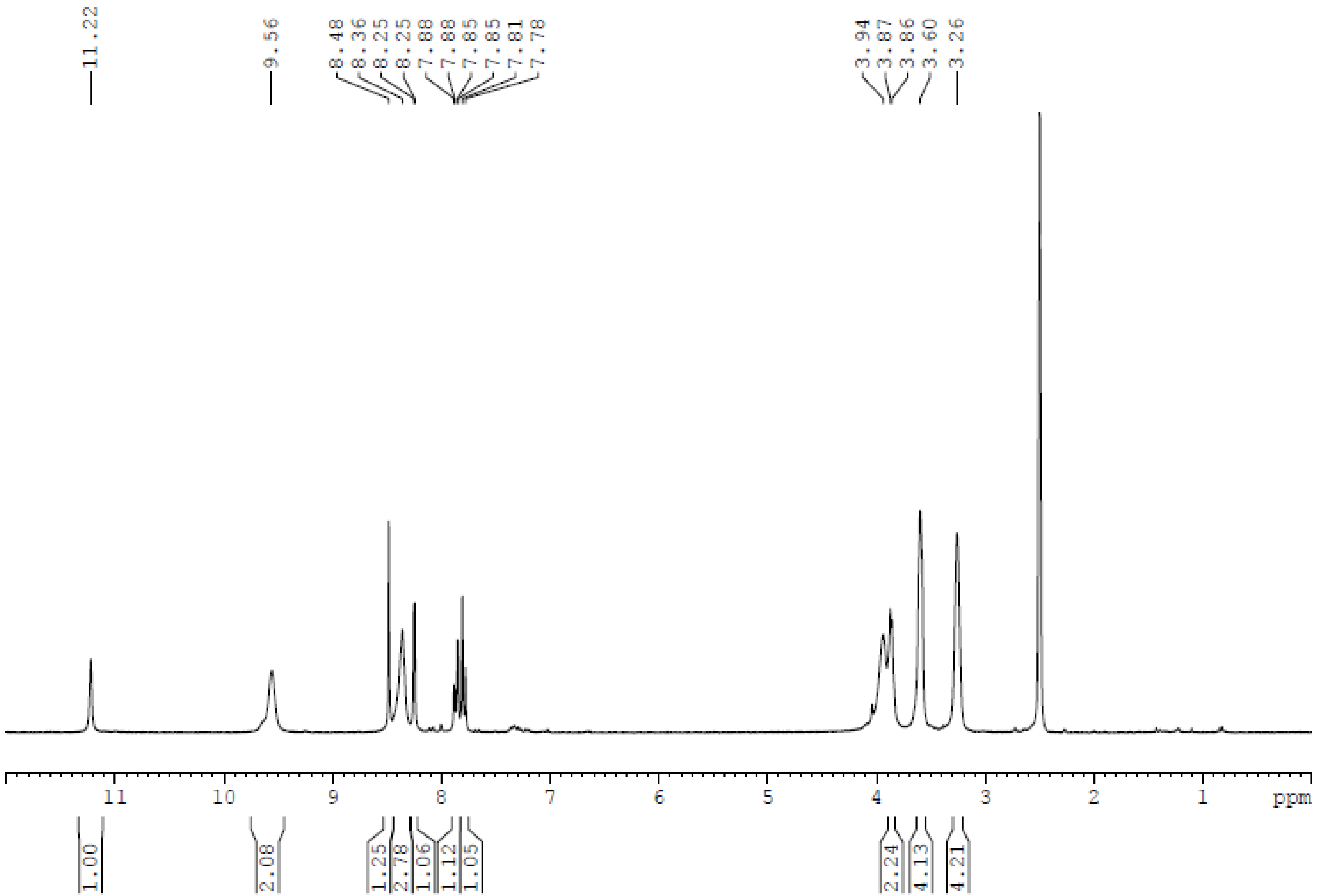

Compound **10** NMR ^{13}C DMSO-d_6

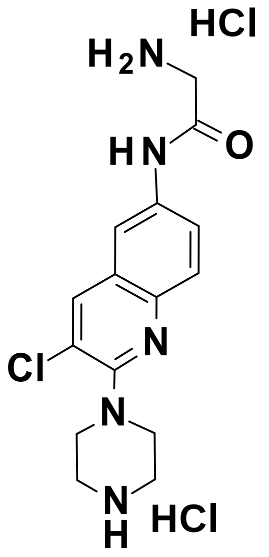

10

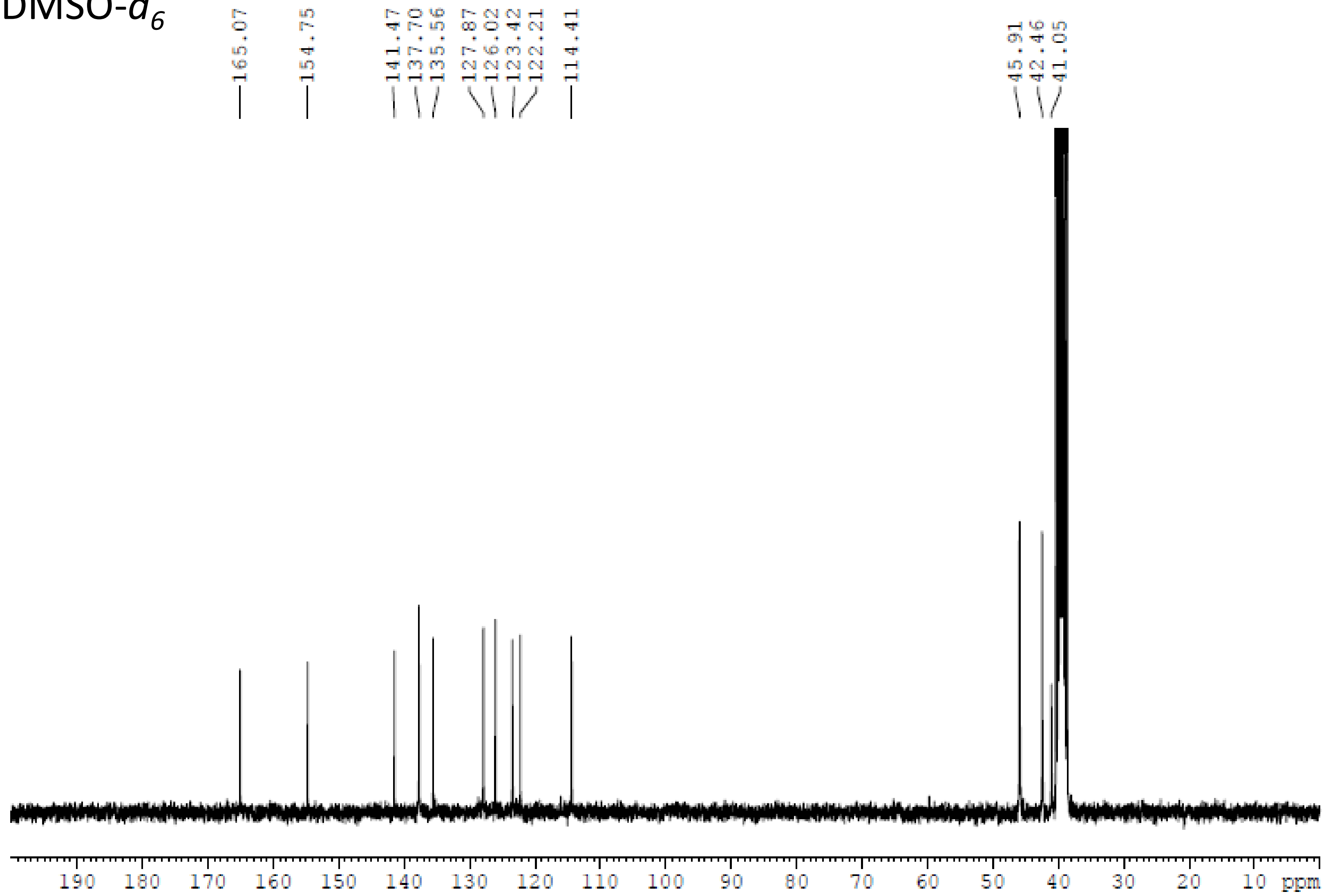

Compound **11** NMR ^{1}H DMSO-d_6

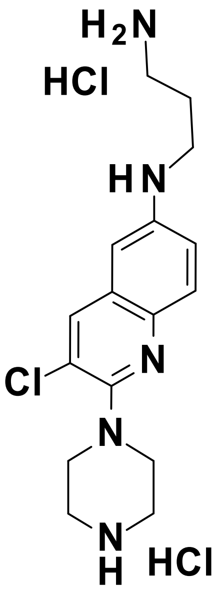

11

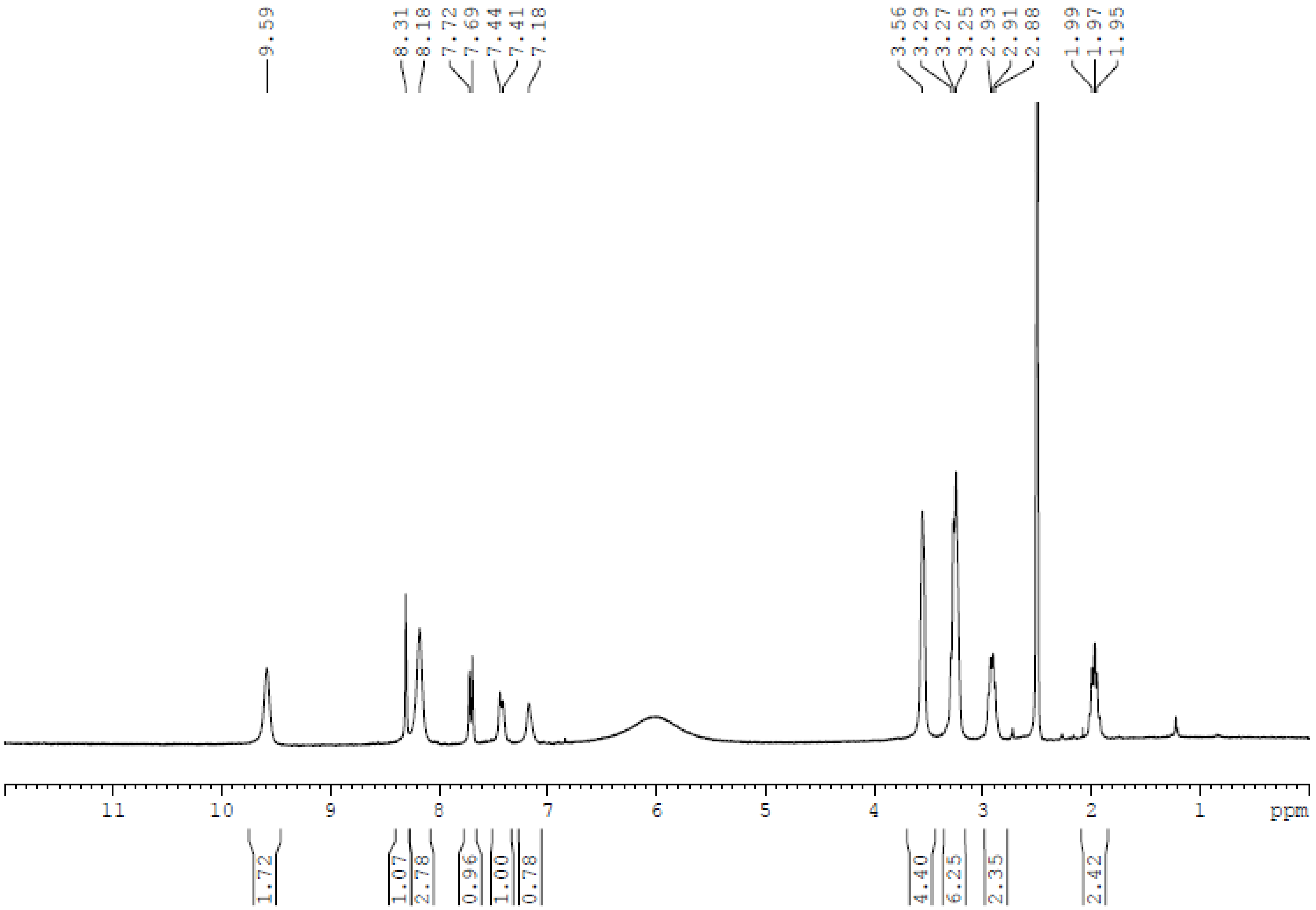

Compound **11** NMR ^{13}C DMSO-d_6

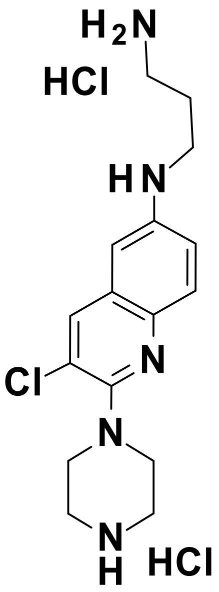

11

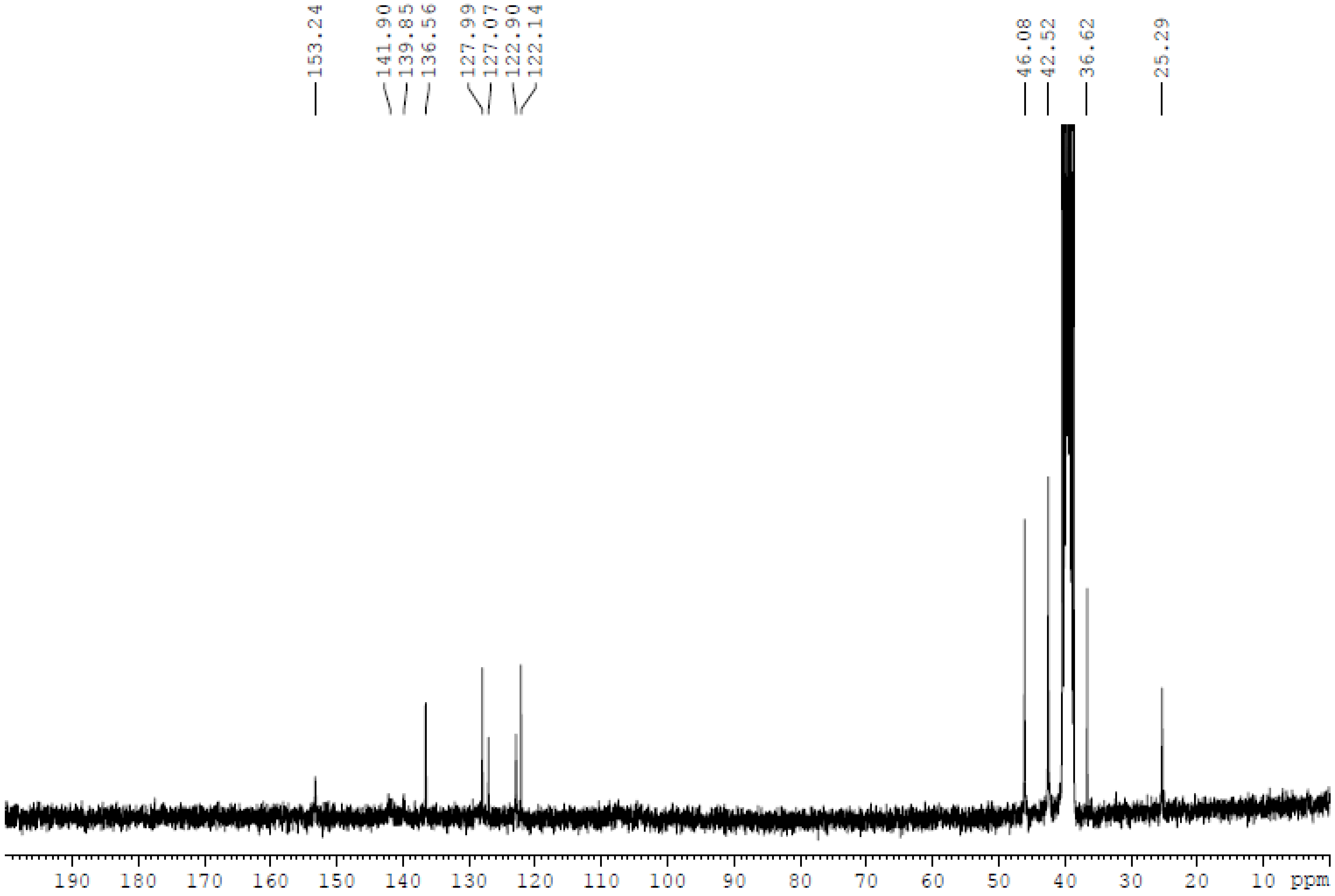

Compound **12** NMR ^{1}H DMSO-d_6

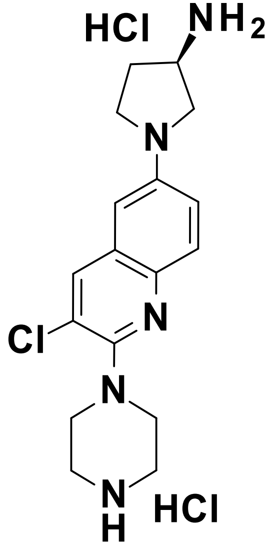

12

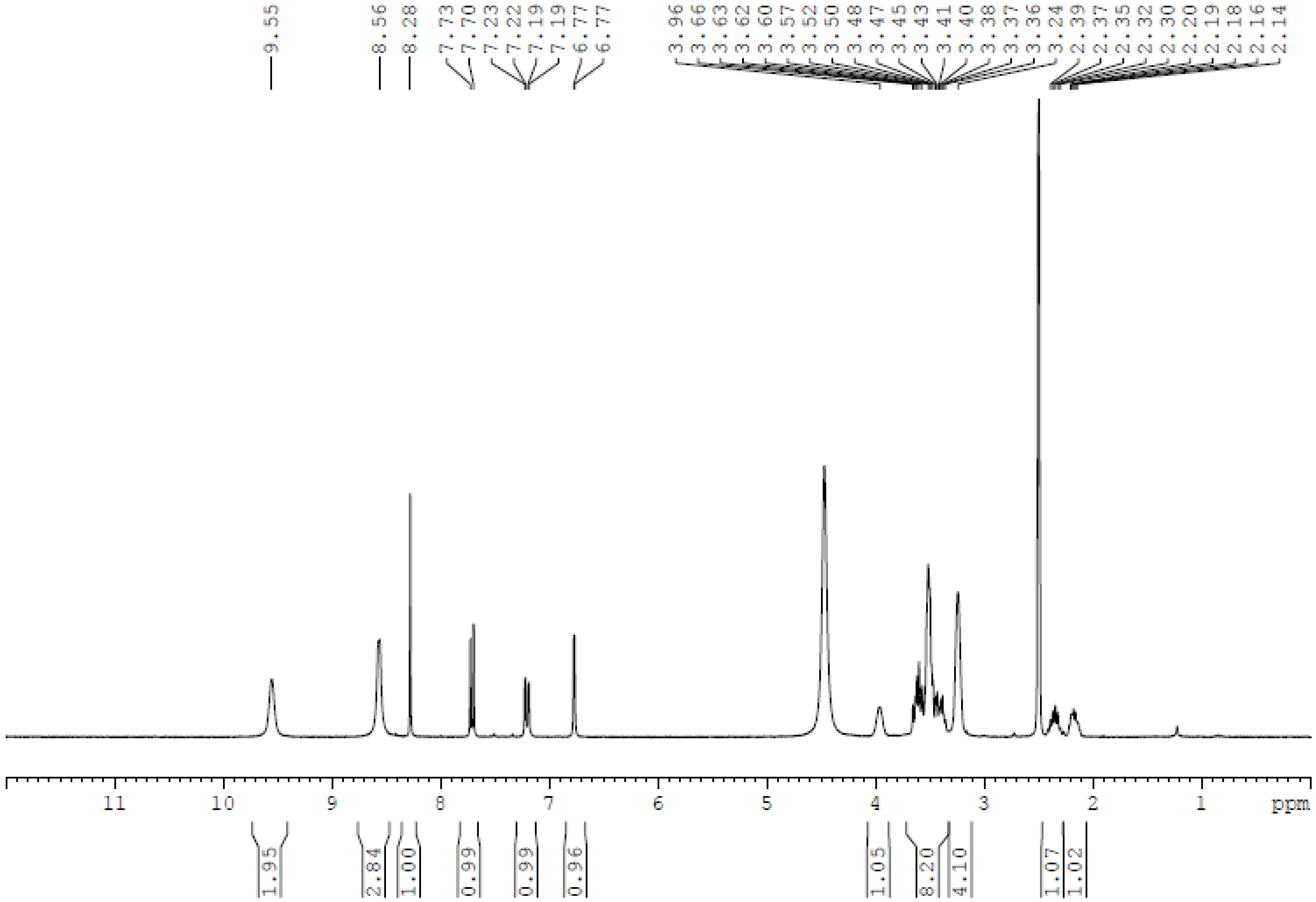

Compound **12** NMR ^{13}C DMSO-d_6

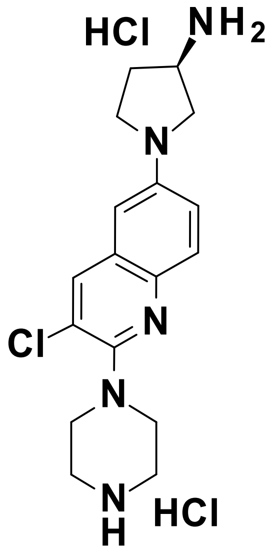

12

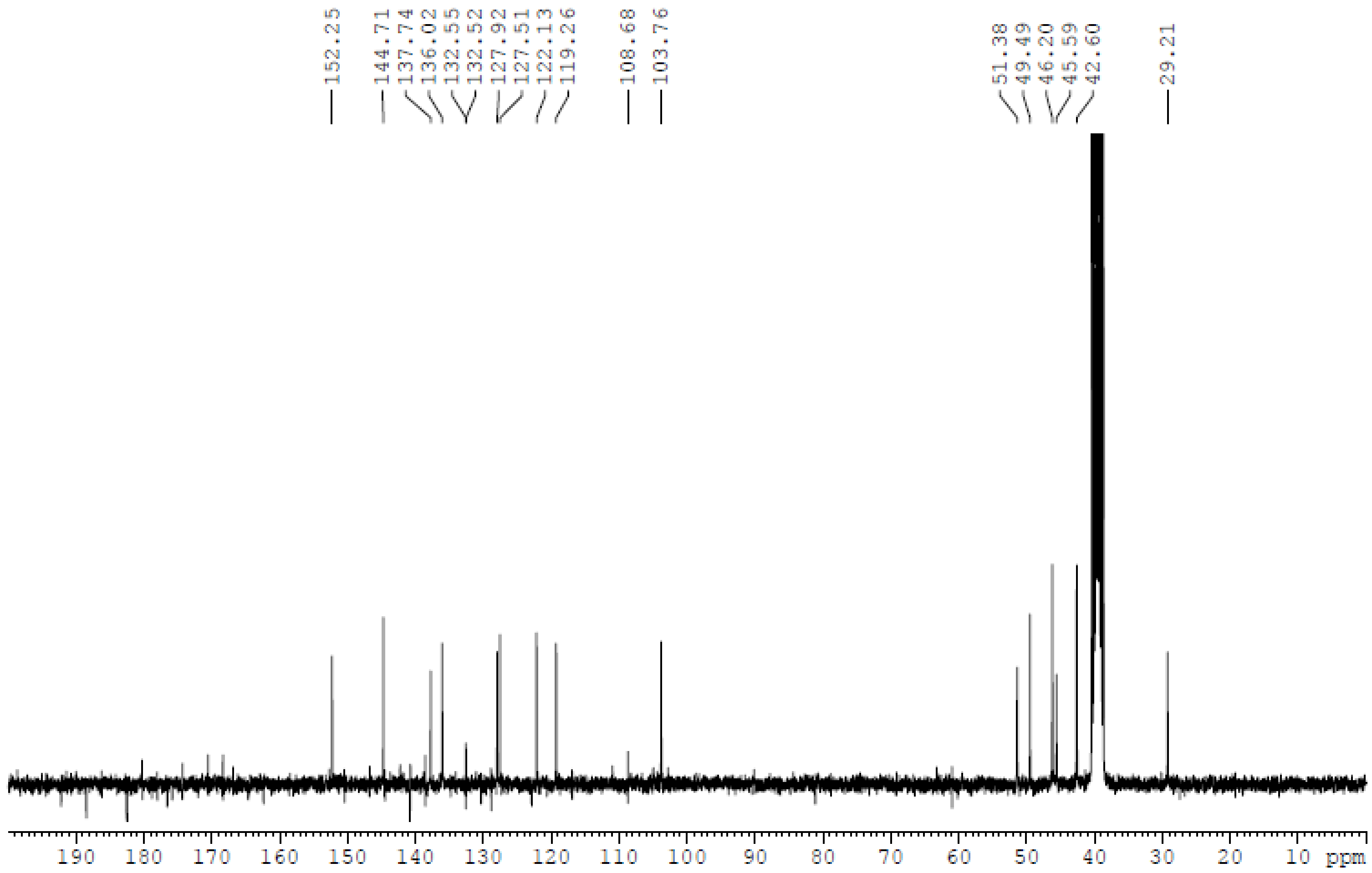

Compound **13** NMR ^{1}H DMSO-d_6

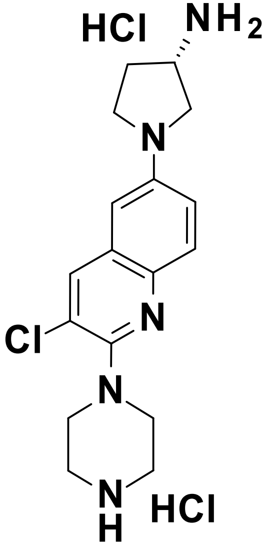

13

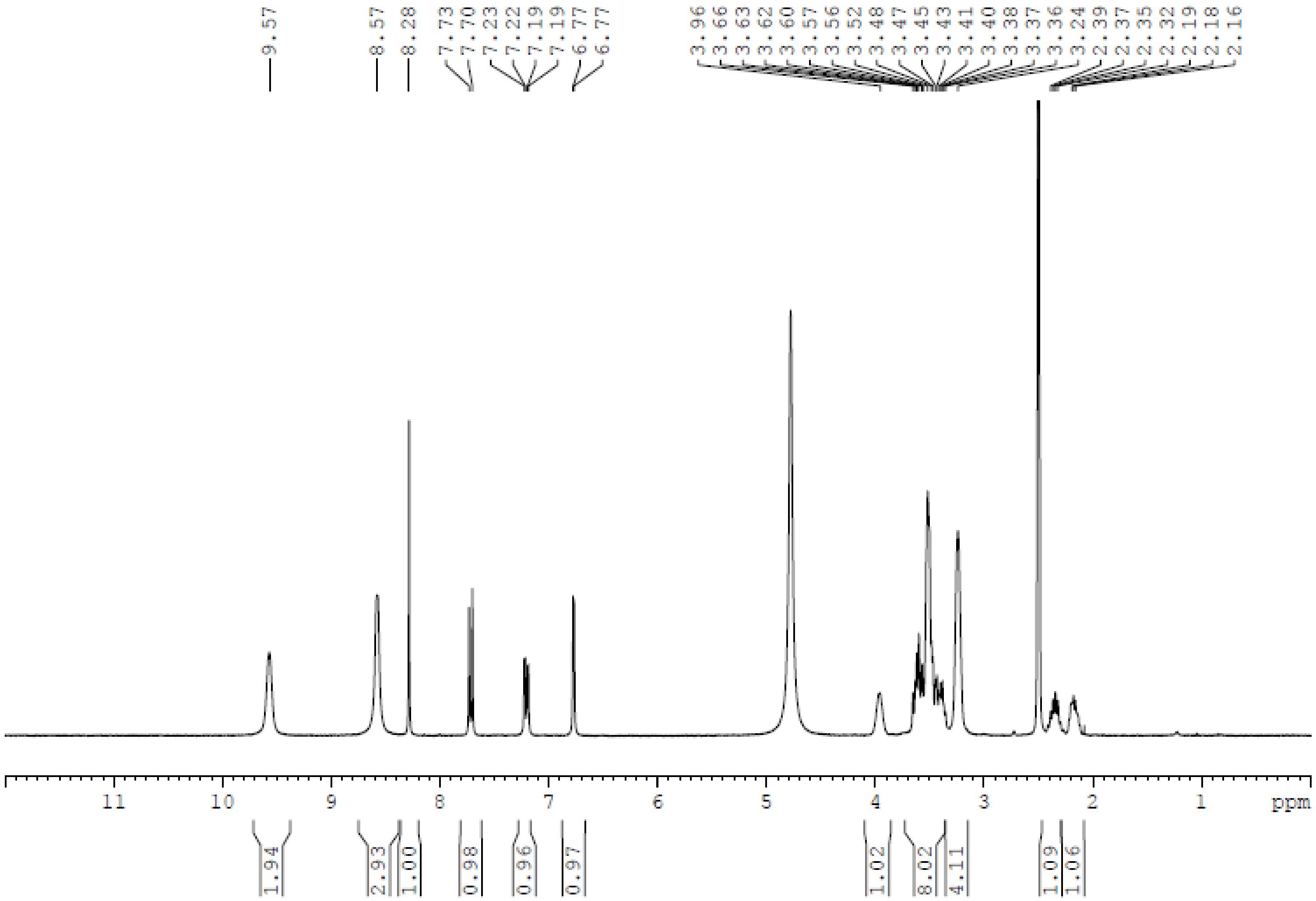

Compound **13** NMR ^{13}C DMSO-d_6

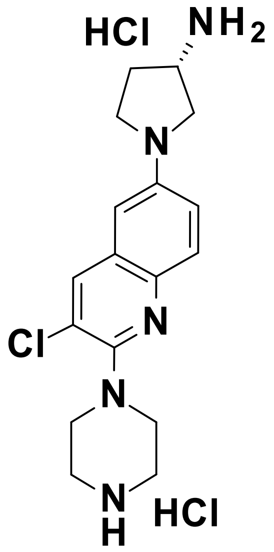

13

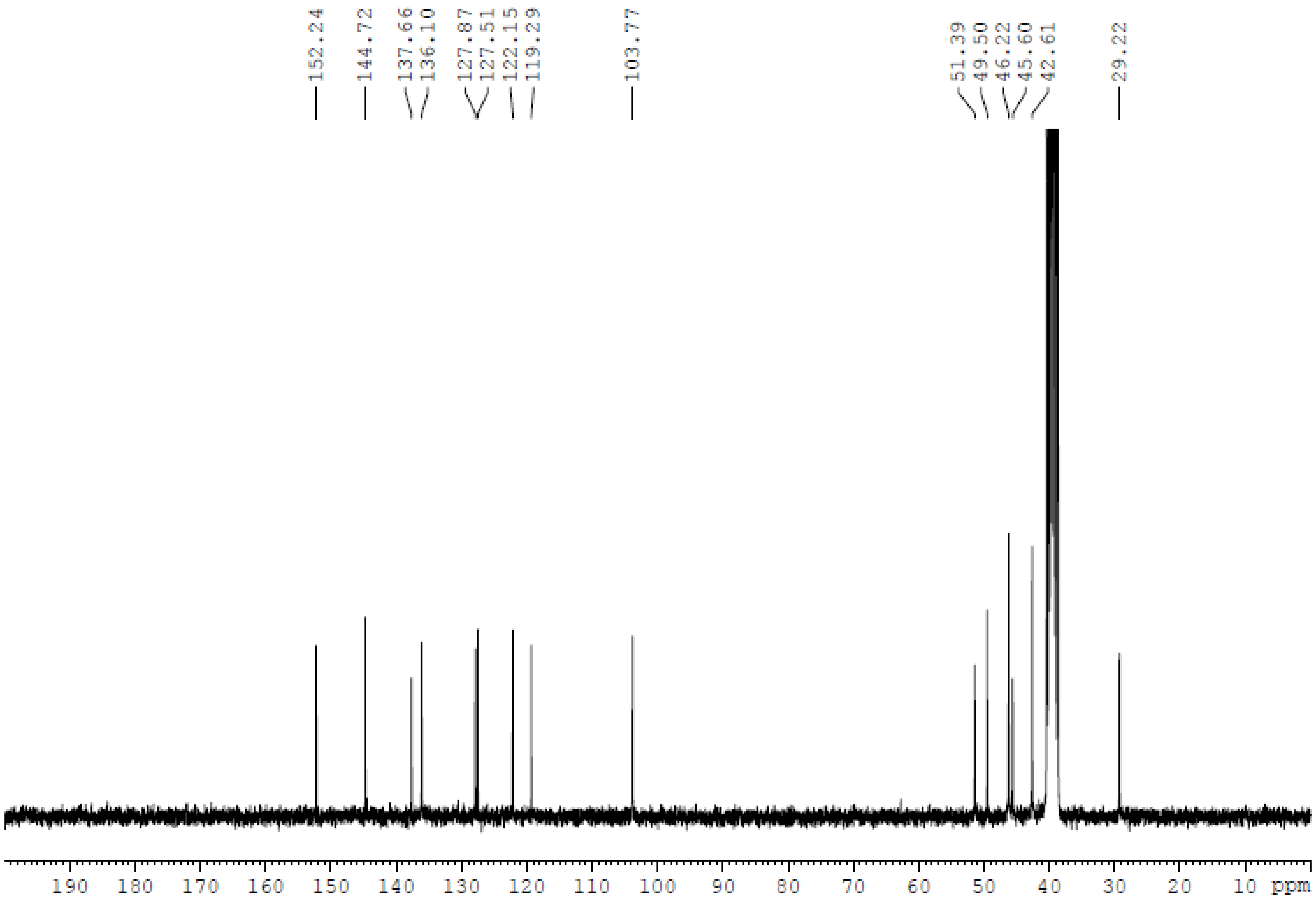

Supplement: Supplementary file 1 — Appendix [file 44321_2023_7_MOESM1_ESM.pdf]
