# Supplementary material for: Pyridylpiperazine efflux pump inhibitor boosts in vivo antibiotic efficacy against K. pneumoniae
Source: EMBO Mol Med. 2023 Dec 20;16(1):93–111. doi: 10.1038/s44321-023-00007-9 (PMC10897476; doi:10.1038/s44321-023-00007-9)
Supplement: Supplementary file 6 — Expanded View Figures [file 44321_2023_7_MOESM6_ESM.pdf]

Expanded View Figures

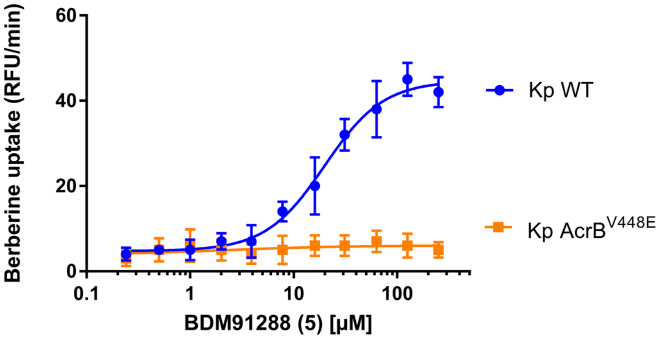

Figure EV1. Rate of berberine uptake by *K. pneumoniae* WT and *K. pneumoniae* AcrB^{V448E} in dependence of increasing BDM91288 (5) concentrations.

Data are a mean and SD of four independent experiments. Source data are available online for this figure.

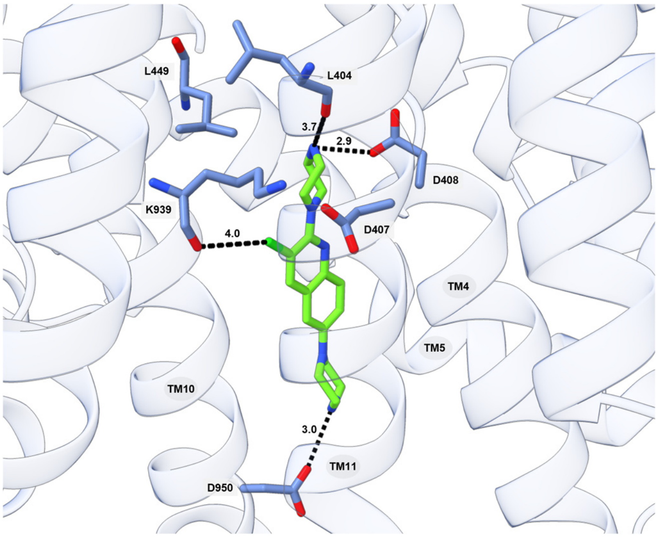

Figure EV2. Structure of *Kp*AcrB in complex with BDM91288 (5).

Enlarged view of the inhibitor binding site showing interacting residues (blue sticks) in less or equal than 4 Å distance from BDM91288 (**5**). The salt bridge between the distal piperazine ring and D408 (TM helix 4), the halogen bond between the BDM91288 (**5**) chlorine and the K939 (TM helix 10) main chain carbonyl oxygen, the hydrogen bond between the L404 (TM helix 4) main chain carbonyl oxygen and the distal piperazine ring, and the salt bridge between D950 (TM helix 10) and the proximal piperazine ring are indicated by dashed lines and numbers represent the distance in Å.

 © The Author(s)

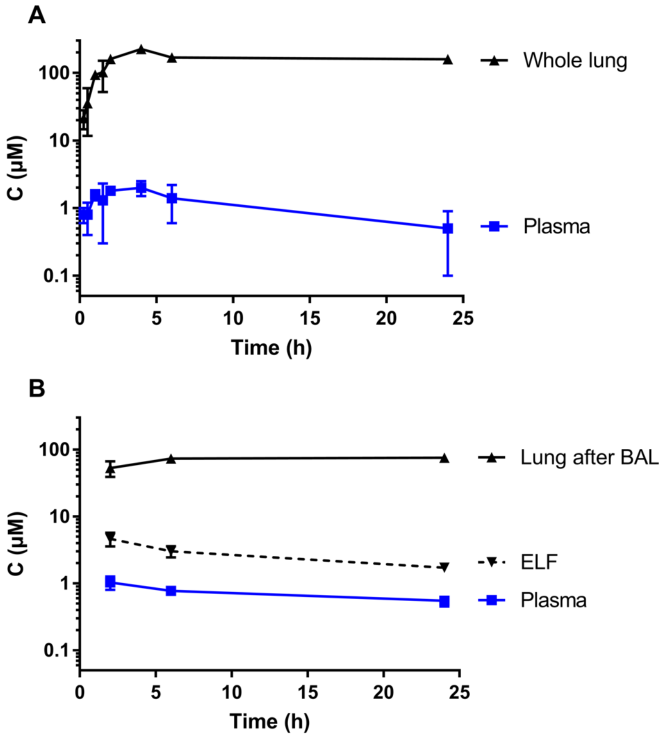

Figure EV3. Pharmacokinetic profile of a single dose BDM91288 (5) (30 mg/kg, orally, formulated in 10% hydroxypropyl-β-cyclodextrin) in mice.

(**A**) Concentration of BDM91288 (**5**) versus time in whole lung and plasma. Plots show the mean ± SD ($n = 3$). (**B**) Concentration of BDM91288 (**5**) versus time in lung after bronchoalveolar lavage (BAL), epithelial lining fluid (ELF) and plasma. BDM91288 (**5**) was given in combination with a single dose levofloxacin (10 mg/kg in water, i.p.). Plots show the mean ± SD ($n = 4$). Source data are available online for this figure.

© The Author(s)
